# Comparison of a robotic-assisted gait training program with a program of functional gait training for children with cerebral palsy: design and methods of a two group randomized controlled cross-over trial

**DOI:** 10.1186/s40064-016-3535-0

**Published:** 2016-10-28

**Authors:** Alicia J. Hilderley, Darcy Fehlings, Gloria W. Lee, F. Virginia Wright

**Affiliations:** 1 Bloorview Research Institute, Holland Bloorview Kids Rehabilitation Hospital, 150 Kilgour Rd, Toronto, ON M4G 1R8 Canada; 2 Rehabilitation Sciences Institute, Faculty of Medicine, University of Toronto, Toronto, ON Canada; 3 Department of Paediatrics, Faculty of Medicine, University of Toronto, Toronto, ON Canada; 4 Department of Physical Therapy, University of Toronto, Faculty of Medicine, Toronto, ON Canada

**Keywords:** Cerebral palsy, Physical therapy modalities, Gait, Robotics, Orthotic devices

## Abstract

**Background:**

Enhancement of functional ambulation is a key goal of rehabilitation for children with cerebral palsy (CP) who experience gross motor impairment. Physiotherapy (PT) approaches often involve overground and treadmill-based gait training to promote motor learning, typically as free walking or with body-weight support. Robotic-assisted gait training (RAGT), using a device such as the Lokomat^®^Pro, may permit longer training duration, faster and more variable gait speeds, and support walking pattern guidance more than overground/treadmill training to further capitalize on motor learning principles. Single group pre-/post-test studies have demonstrated an association between RAGT and moderate to large improvements in gross motor skills, gait velocity and endurance. A single published randomized controlled trial (RCT) comparing RAGT to a PT-only intervention showed no difference in gait kinematics. However, gross motor function and walking endurance were not evaluated and conclusions were limited by a large PT group drop-out rate.

**Methods/design:**

In this two-group cross-over RCT, children are randomly allocated to the RAGT or PT arm (each with twice weekly sessions for eight weeks), with cross-over to the other intervention arm following a six-week break. Both interventions are grounded in motor learning principles with incorporation of individualized mobility-based goals. Sessions are fully operationalized through manualized, menu-based protocols and post-session documentation to enhance internal and external validity. Assessments occur pre/post each intervention arm (four time points total) by an independent assessor. The co-primary outcomes are gross motor functional ability (Gross Motor Function Measure (GMFM-66) and 6-minute walk test), with secondary outcome measures assessing: (a) individualized goals; (b) gait variables and daily walking amounts; and (c) functional abilities, participation and quality of life. Investigators and statisticians are blinded to study group allocation in the analyses, and assessors are blinded to treatment group. The primary analysis will be the pre- to post-test differences (change scores) of the GMFM-66 and 6MWT between RAGT and PT groups.

**Discussion:**

This study is the first RCT comparing RAGT to an active gait-related PT intervention in paediatric CP that addresses gait-related gross motor, participation and individualized outcomes, and as such, is expected to provide comprehensive information as to the potential role of RAGT in clinical practice.

*Trial registration* ClinicalTrials.gov NCT02196298

**Electronic supplementary material:**

The online version of this article (doi:10.1186/s40064-016-3535-0) contains supplementary material, which is available to authorized users.

## Background

The maintenance and enhancement of walking abilities is an important focus of rehabilitation for children with cerebral palsy (CP) in order to promote the physiological, functional and social benefits associated with ambulation (Stuberg 1992; Eisenberg et al. 2009; McKeever et al. 2013). CP is the most common cause of childhood physical disability (Oskoui et al. 2013) involving heterogeneous motor impairments caused by damage to the fetal or developing brain. Approximately 50% of children with CP require only orthoses or minimal assistive devices for independent mobility (i.e., Levels I and II of the Gross Motor Function Classification System [GMFCS]), while those in GMFCS Levels III and IV need extensive bracing and walkers or wheelchairs to move short and/or long distances (Palisano et al. 2008). Moreover, while the neurological damage is non-progressive in CP, walking skills tend to degrade with growth and age for those in GMFCS levels III and IV (Hanna et al. 2009) leading to increased reliance on non-ambulatory mobility options for efficiency and ease (Bottos and Gericke 2003).

Although wheelchair use does not necessarily lead to physiological or anatomical deterioration (Bottos et al. 2001), the benefits of standing and walking may include enhanced cardiovascular fitness (Park et al. 2001; Bjornson et al. 2007), functional gains (Strifling et al. 2008; Eisenberg et al. 2009) and greater involvement in social roles (Lepage et al. 1998). Furthermore, decreased standing time reinforces sedentary behaviours in children with CP (Verschuren et al. 2014), potentially increasing risk of comorbidities and premature mortality as seen in adults (Peterson et al. 2013). Beyond the health benefits, many families and clinicians emphasize walking because of the dominant societal beliefs that walking holds symbolic value associated with ‘normalcy’ and reduction of the social stigma of disability (Gibson et al. 2012). Combined with the evidence of positive health outcomes, these strong values result in extensive use and development of physiotherapy (PT), orthopaedic and medical interventions that focus on walking (Novak et al. 2013). However, the recent emergence of technologically-based walking interventions has been criticized because of the increased focus on ‘normalcy’ (Phelan et al. 2014), highlighting that persistent efforts towards walking may limit time for other childhood activities and not increase participation (Wiart 2011). In light of these pros and cons, it is important that rehabilitation practitioners seek better understanding of the impact of gait therapies and families’ values related to walking, especially as compelling high technology options such as RAGT become more available (Phelan et al. 2014).

Over the last decade, therapy emphasis for children with CP has shifted from being largely impairment-based (i.e., strength training and range of motion work) to focusing on activity and participation, with incorporation of motor learning principles (Park and Kim 2013). These principles include high repetition and active participation of the learner to promote neuroplasticity and skill acquisition (Levac et al. 2009). In the context of walking-focused therapies, technology can capitalize on motor learning principles (Schindl et al. 2000). For example, partial body-weight supported treadmill training (PBWSTT) systems extend the opportunity for repetitive gait training to children who have lower tolerance for independent ambulation (GMFCS Levels III and IV) (Palisano et al. 2008), supporting a focus on quality of gait and walking experience (motor learning aspects), as well as on walking endurance. Although PBWSTT results in improvements of time-distance aspects of gait and gait-related functional abilities for children in GMFCS II–IV (Willoughby et al. 2009; Zwicker and Mayson 2010; Bar-Haim et al. 2010; Johnston et al. 2011; Chrysagis et al. 2012), the extensive manual assistance required for those in GMFCS III–IV often makes it difficult to use. Functional electrical stimulation (FES) devices can be used to stimulate muscle activation, however these devices typically target one muscle to support movement of one joint (Pool et al. 2014). Many children with CP require multi-joint assistance for repetitive gait training. Robotic-assisted gait training (RAGT) devices, such as the commercially available Lokomat^®^Pro (Hocoma AG, Switzerland, www.hocoma.com), were designed to address these physical limitations through use of robotic leg orthoses to guide leg movement, and have been reported to be at least as efficacious as manually assisted PBWSTT (Tefertiller et al. 2011).

There is strong evidence of improved functional outcomes following RAGT in adults with spinal cord injury and stroke, including increased gait speed and endurance in the 6-minute walk test (Tefertiller et al. 2011), but gains are not always superior to traditional PT outcomes (Dobkin and Duncan 2012). In children and youth with CP, knowledge about the impact of RAGT is limited (Castelli 2011). One-group studies have demonstrated an association between RAGT training and moderate to large improvements in gross motor skills, gait velocity and endurance (Meyer-Heim et al. 2007, 2009; Koenig et al. 2008; Brütsch et al. 2010; Drużbicki et al. 2010; Borggraefe et al. 2010a, b; Pajaro-Blazquez et al. 2013; Schroeder et al. 2014b) as well as improvements in performance and satisfaction of activities of daily life/participation (Schroeder et al. 2014a, b). A single published randomized controlled trial (RCT) comparing Lokomat training to a PT-only intervention showed no difference in gait kinematics, yet gross motor function and walking endurance were not evaluated and conclusions are further limited by a greater than 50% drop-out rate in the control group (Druzbicki et al. 2013). At the time of designing this study and receiving funding, only one RCT comparing Lokomat training to a waitlist control group was active (clinicaltrials.gov). A summary of the paediatric studies and key results is provided in Additional file 1.

Stronger evidence is required to provide the necessary understanding of the role and potential of RAGT as a clinical therapy for children with CP (Zwicker and Mayson 2010; Druzbicki et al. 2013; Aurich Schuler et al. 2015). To address this knowledge gap, we developed an RCT with a cross-over design to compare a program of RAGT using the Lokomat to a gait-related PT program in children with CP in GMFCS Levels II and III. These children are expected to have primary goals that are walking-based, and their existing ability to ambulate permits use of gait-related therapeutic content in each intervention arm. The chosen outcome measures extend beyond the GMFM and assessment of gait, and incorporate individualized goals as well as activity and participation outcomes. By controlling for concurrent PT, co-intervention concerns arising from previous studies of RAGT are reduced. Further, an acknowledged limitation of RAGT is that participants can passively move through a standardized gait cycle (Koenig et al. 2011) and if sessions are not designed to be interactive, the benefits may be inferior to an active PT program. Thus both Lokomat and PT intervention protocols used in this study are grounded in motor learning theory, following principles to optimize learning and promote active engagement of the participant by progressively increasing the challenge, introducing variability, promoting high repetition, and practicing activities that are meaningful to the child by specifically targeting the child’s goals (Levac et al. 2009). Intervention protocols are fully operationalized and will be reported in detail (a critical aspect of external validity) to provide clinically applicable information not included within Lokomat study publications thus far.

## Methods

**Fig. 1:**
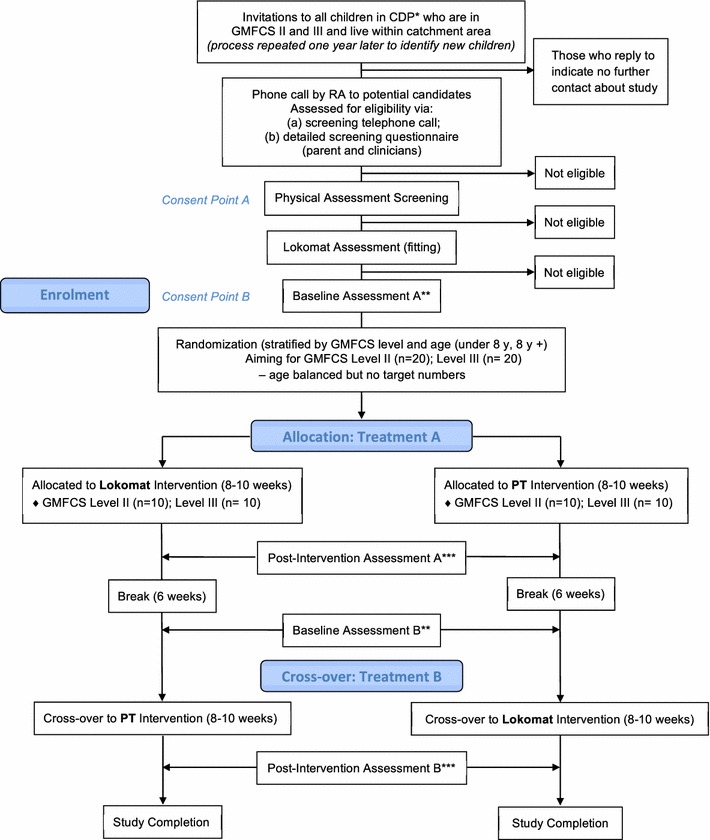
Flowchart of enrolment according to CONSORT guidelines. *CDP: Child Development Program at Holland Bloorview Kids Rehabilitation Hospital, Toronto, Canada. **Baseline Assessments occur <14 days prior to start of intervention arm. ***Post-Intervention sessions occur <7 days after end of intervention arm

The study is a two group (Lokomat intervention versus gait-related PT intervention), randomized cross-over trial (Senn 2002) with a six-week break between the two intervention arms (A and B) at the time of cross-over. Assessments are done at four time points: baseline pre-intervention A, post-intervention A; baseline pre-intervention B, and post-intervention B. CONSORT (Schulz et al. 2010) and SPIRIT (Chan et al. 2013) guidelines were taken into account in the design of the trial protocol, and the CONSORT diagram (modified for cross-over trials) is provided in Fig. 1. Assessments and intervention sessions are all conducted at Holland Bloorview Kids Rehabilitation Hospital, Toronto, Canada. Full ethical approval was obtained from the Research Ethics Board (REB) at Holland Bloorview. This REB must approve any potential protocol amendments, and regulate whether amendments must be communicated to participants and families.

Further details and rationale for the design decisions are provided in the “Design” section below.

## Study aims and hypothesis

The primary aim of the study is to determine the impact on gait-related gross motor skills of a gait training program using a robotic-assisted gait trainer (the Lokomat^®^Pro) compared with a gait-related physiotherapy (PT) program for children in GMFCS Levels II and III. The primary hypothesis is that children receiving the Lokomat intervention will improve more on the GMFM-66 and 6-minute walk test (6MWT) than those in the gait-related PT program.

The secondary objective is to evaluate the comparative impact of Lokomat training and gait-related PT on: (a) individualized walking/gross motor based goals; (b) the amount/location of walking the child does each day (environmental context and season considered); (c) participation in activities and (d) health-related quality of life.

## Study sample and recruitment

The study aims to enrol 40 children with CP (ages 5–12 years) as follows: (a) 20 children in GMFCS Level II who have walking limitations but ambulate without a gait device or crutches or canes for most distances; and (b) 20 children in GMFCS Level III who have a greater extent of walking limitation and use walkers or wheelchairs for short to long distances (Russell et al. 2000).

### Inclusion criteria

Children are: (a) age 5 to12 years inclusive; (b) in GMFCS Levels II or III; (c) able to follow testing instructions, and participate in a minimum of 30 min of active PT; (d) able to reliably signal pain, fear and discomfort; (e) have passive range of motion (ROM) of hips and knees within minimum range requirement for Lokomat (hip and knee flexion contracture ≤10°, and knee valgus ≤40°); and (d) able to commit to attendance of twice weekly for eight weeks (to support the primary efficacy analysis).

### Exclusion criteria

In addition to all relevant criteria outlined in the Lokomat manufacturer’s manual (Hocoma AG, Switzerland, www.hocoma.com), children are excluded if they have: (a) hip instability/subluxation >45%; (b) orthopaedic surgery within the last 9 months (muscle) or 12 months (bone); (c) Botulinum toxin-A (BTX-A) injections to lower limb in the last 3 months; (d) inability to discontinue BTX-A for period of 6 months (during trial) due to concerns about ROM or pain; (e) any weight bearing restrictions; (f) seizure disorder that is not controlled by medication (if on medication, must not have had a seizure in the last 12 months); (g) open skin lesions or vascular disorder of lower extremities; or are: (h) not able to co-operate or be positioned adequately within the Lokomat as shown during the two Lokomat fitting/acclimatization sessions; (i) not prepared or unable to discontinue a regular therapy intervention during the course of the trial; (j) involved in another intervention study.

## Design

This study uses a cross-over design to compare the *two* different therapy intervention programs: robotic assisted gait training (Lokomat) versus gait-focused PT (see Fig. 1). The order of treatment (Lokomat or PT first) is designated by random assignment. Children cross-over to the second intervention arm following a six-week non-treatment break between interventions. The second intervention arm begins after a second baseline assessment completed at the end of the break to re-establish the child’s abilities. The use of a cross-over design helps to reduce the impact of confounding variables outside of the control of the study itself, since each cross-over participant acts as his/her own control (i.e., reduces between subject variation) (Senn 2002). Cross-over designs are also more efficient than standard RCTs or repeated measure designs, requiring fewer participants (Louis et al. 1984). While there may be concern that the extended length of involvement in the trial to complete both phases may be an obstacle for enrolment or adherence, cross-over designs have been successfully employed in CP and Lokomat research (McNee et al. 2007; Mayr et al. 2007). Furthermore, the opportunity to receive both intervention arms and be assured access to the Lokomat is thought to outweigh any perceived burden of the time commitment (Law et al. 1997).

Each intervention is given twice weekly for a total of 16 sessions over a targeted treatment period of 8 weeks. This training frequency mirrors a standard clinical PT treatment block. A maximum of 10 weeks is targeted to keep the dose as close to twice a week as possible while taking into account the occasional need to miss a session due to illness, vacation, and other unavoidable interruptions. The total number of sessions is similar to trials in which Lokomat effectiveness was demonstrated (Koenig et al. 2008; Borggraefe et al. 2010a, b; Meyer-Heim and van Hedel 2013; Schroeder et al. 2014a, b) while a chief difference is the total duration of the treatment period, in that other Lokomat trials have used a short intensive burst of therapy (e.g., 15 treatments over three weeks). Our concern from a motor learning and assimilation standpoint is that a short burst may be insufficient time for the child to develop and integrate new skills gained through training into their daily life.

Children are required to discontinue regular PT interventions prior to the first baseline assessment and to abstain from starting any other PT during the trial to reduce the chance of confounding effects associated with any previous or concurrent treatment. The child is allowed to continue any existing program of soft tissue stretches and/or basic walking and standing, but their clinical physiotherapist (PT), or other clinician, is asked not to alter the programs during the course of the study. Activities are monitored at the start of each intervention session (Lokomat or PT), when the child and parent are asked by the study PT about any other gross motor-based treatments and physical activity they did since the last session. These physical activity descriptions are documented in the child’s session log. The study PT is required to alert the research assistant (RA) to any potential co-interventions. Subsequently, the RA calls the parent to ask them to put any activities on hold that are judged to be walking-related co-interventions.

During the six-week break that occurs before crossing to the other intervention arm, the child is allowed to continue their existing regimen of soft tissue stretches and any basic walking/standing home program they had been given prior to the trial, but asked not to embark on any other new therapy. The RA phones the parent mid-way through this phase to check for any treatments that the child may have started/resumed. A request to discontinue any added treatment may be made by the RA after discussion with the co-Principal Investigators (co-PIs) if it appears to be a gait-related co-intervention (i.e., a possible confounder). While the six weeks between the two intervention arms is not a true wash-out period (since effects of rehabilitation are not expected to be reversible when therapy is withheld), it does give a time break for families between the two phases. A new baseline is established before starting the second intervention to ensure intervention effects are isolated for analysis (Mayr et al. 2007).

### Study enrolment

Invitation letters are sent to parents of eligible participants registered in the Child Development Program (CDP) at Holland Bloorview Kids Rehabilitation Hospital, a program with a large CP population that will support achievement of target enrolment numbers. If interested, parents complete a basic screening over the phone with the RA followed, if suitable, by a more detailed eligibility questionnaire. An eligibility form is also sent, with parents’ permission, to the child’s clinicians to ensure there are no concerns with study participation and use of the Lokomat. The second step for children who meet these initial criteria is a formal screening assessment. The co-PIs then review findings with the assessor and confirm with the family whether the child is eligible for the study based upon these findings (see Fig. 1 for details). The RA obtains written informed consent from participants and parents at two time points: prior to study screening assessment and, if screening criteria are satisfied, prior to participating in the intervention. Assent is obtained from children incapable of providing independent consent.

### Randomisation

Children who meet the eligibility criteria and pass the screening assessment are assigned a study ID number, and then attend their baseline assessment. Following the baseline assessment, the child is allocated to start in either the Lokomat or PT intervention phase via a process of random assignment using a computer generated random numbers system. The assignment scheme was prepared at the start of the study by a non-study RA, using a stratified randomisation process (age and GMFCS level) that employed mixed allocation blocks of 4 and 6 children. By the end of the trial, this system will ensure that the number and age/GMFCS balance of participants who start in the Lokomat and PT intervention groups will be even. Management of the randomisation process by a non-study RA through the entire study allows the study investigators to remain blinded to the scheme, thereby eliminating allocation bias. Participants are informed of the intervention order by the study RA.

### Blinding

It is not possible to blind the parent or child or study PT to the study interventions. The study’s PT assessors (a separate group from the treating study PTs) are blinded to the child’s treatment arm. The same assessor evaluates the child at each of the baseline and follow-up sessions. The inability to guarantee full success of the blinding is due to overlapping clinical and research treatment/assessment areas, and the possibility that despite asking them not to, children and parents might inadvertently comment on their treatment session experiences to the assessor. Given the strength of objectivity of the outcome measures at single points in time however, concern about the impact of knowledge of group membership is low (Wood et al. 2008). More importantly, to prevent any influence of knowledge of previous results on current assessments, prior assessment data are not available to the assessor at any of the follow-up assessments. Finally, to verify unbiased scoring on the key observational measures (GMFM-66, 6MWT, *Challenge* Assessment, Timed Up and Go), a non-study RA will select a random sample of 20% of the assessment video-recordings to be scored by an independent PT assessor blinded to the assessment’s order in the evaluation sequence.

The study statistician is blinded to group allocation in all analyses. All data are stored in a secure database using Research Electronic Data Capture (REDCap) (Harris 2012), and are entered by the study RA. Data are de-identified using participant study codes. Access codes will be given to the co-PIs for select data sections (i.e., demographics, individualized goals [for purposes of fidelity monitoring], treatment adherence, adverse event tracking), but lack of access to other outcome areas of the database supports PI blinding to the results during the trial. This is important to keep study communications and decisions free from the influence of knowledge of outcomes.

## Study interventions

In both the PT and Lokomat intervention arms, the study PTs have the opportunity to look at the baseline assessment data acquired prior to the first Lokomat or PT session to ensure sufficient clinical understanding of the child to be able to make suitable treatment and goal decisions. Within each intervention arm, a study treatment log of each session is kept for every participant. Each session is fully documented by the study PT on log sheets that require detailed and open disclosure of all activities performed. This log is available to the study PT at each session to permit reference back to previous sessions and support progression. The logs are checked at bi-weekly intervals by the study RA so that any fidelity issues can be addressed in subsequent sessions. Following completion of the intervention arm, completed forms are removed and replaced by a new set of treatment logs. The study RA will also monitor session attendance to problem solve any issues promptly.

The information recorded in these session logs will allow us to fully operationalize all of the aspects of the treatment approaches at the end of the study (an important external validity consideration). The within-session documentation process is also expected to optimize adherence of the study PT to the intervention protocols (treatment fidelity). Furthermore, two of the child’s Lokomat and PT sessions are filmed to permit evaluation at the end of the study of the extent and type of motor learning strategy use within the interventions, using the Motor Learning Strategy Rating Instrument (MLSRI) developed by Levac et al. (2009), and validated by Kamath et al. (2012) at our centre.

### Lokomat intervention protocol

At the start of the child’s Lokomat intervention block, there are two initial fitting/acclimatization sessions, and then 16 twice-weekly Lokomat sessions over an 8- to 10-week period. Fitting the child and acclimatization on the Lokomat is done as outlined in the Lokomat User Manual (Hocoma AG, Switzerland, www.hocoma.com). The goal is to ensure that the child is comfortable in the exoskeleton (e.g., right fit of straps and cuffs, alignment set within a tolerable range of movement) as well as on the treadmill mechanism, and that the child is able to follow the PT’s instructions and to indicate any discomfort. The Lokomat intervention is designed as a standardized protocol, and the content and method for progressing settings on the Lokomat are outlined in Additional file 2. The home program component is limited to basic stretching, strengthening exercises and/or walking practice that the child was on pre-study.

The Lokomat treatment sessions start with 10–20 min on the Lokomat, increasing as tolerated within three sessions to the 30 min maximum target time for this study (Mayr et al. 2007). Four parameters are adapted to the ability, strength, and endurance of the child: body weight support provided; walking duration; ambulation velocity; and guidance force provided to each leg. Guidelines for parameter adjustments are provided in Additional file 2 along with suggested protocols in the Lokomat User Manual, which will inform the setting changes the therapist makes throughout the session. These adjustments will be made to target the child’s gait goals. If a child is unable to keep up with any of the progressions, adjustments will be carried out to a lesser extent or reversed. From a content perspective, the motor-learning based protocol follows both the Lokomat guidelines for encouraging lower limb selective muscle/joint activation and motor learning, and also includes a listed series of upper body tasks that can be done while walking (e.g., throwing a ball, hitting an overhead object) to encourage multi-tasking and improved posture. Use of Lokomat virtual reality games and provision of visual biofeedback are also permitted to promote engagement and feedback. Each session is followed by 5 min of rest before a 5-min overground gait training session to reinforce the Lokomat session gait focus outside of the Lokomat.

Information on the body weight support provided, walking duration, ambulation velocity, and guidance force provided to each leg are recorded in the child’s treatment log as they occur during the session along with details of any breaks or “free rides” (time spent passively) in the Lokomat. In keeping with the principles of motor learning, the goal is to optimize active participation and keep passive walk time to a minimum. The Lokomat computer also continuously tracks all of the settings used throughout the session and provides setting-based information related to any safety stops initiated by the Lokomat. Safety stops occur in reaction to movement forces from the child that are outside of the parameter boundaries of the Lokomat for that child.

### Gait-related PT intervention protocol

This intervention has been created as a motor-learning outpatient program that requires intensive PT guidance, coaching and therapy equipment. At the start of the child’s PT intervention block, there is one acclimatization session to introduce the child to the PT gym equipment and surroundings, and then 16 twice-weekly PT sessions over an 8- to 10-week period. Each PT session consists of 35 min of active treatment with 10 additional minutes of introduction and wrap-up conversation, which parallels the 30 min of Lokomat walking, 5 min of overground walking and similar period of conversation times in the Lokomat group.

The PT session follows a manualized protocol created specifically by Holland Bloorview Child Development Program PTs in conjunction with study co-PIs (see Additional file 3). This GMFCS level-specific PT program is grounded in current evidence-informed clinical practice. From a content perspective, it is menu-based with delineation of key categories of treatment focus. Treatment components are provided within each of these categories. Virtual reality (VR) interventions are permitted as these are now a standard part of PT treatment (Wang and Reid 2011). Treadmill training is allowed to a maximum of 10 min, but body weight support is not permitted as per design in Mayr et al. (2007). This avoids potential contamination through introducing elements of the weight-relieving and limb guidance gait component of the Lokomat protocol into the PT intervention. Other interventions to be avoided are those that are outside mainstream PT (e.g., Cuevos Medek, conductive education techniques) or focus on changes to body structures (e.g., lower limb Botox, inhibitive casting, osteopathy, kinesiotaping).

The PT is at liberty within each session to choose treatment categories and components that link with the child’s goals (as documented at the start of the intervention phase) and progress those goals as they see appropriate based on their clinical judgment. Each component that is given is recorded in the child’s treatment log at the end of the session along with the area of primary focus of the activity (chosen from a list of areas that include balance, strength, agility, gait quality, walking endurance, etc.), details of repetitions, equipment use or settings, and number of minutes spent on each activity. As with the Lokomat arm, the home program is limited to the basic stretching and strengthening exercises and walking practice that the child was on before they entered the study. For children who start in the PT arm, this will help to reduce the possibility of carryover of home treatment ideas from the PT intervention into the Lokomat phase (contamination).

### Study intervention PTs

There are 12 paediatric PTs (all specialists in working with children with CP or brain injury) who were specially trained as described below to provide the Lokomat and PT interventions. Each child is assigned to a study treatment team consisting of two PTs who share responsibility for the twice-weekly treatment sessions. Use of a two-member intervention team is consistent with typical models of service delivery in which a PT and PT Assistant (PTA) share responsibility for a child’s treatment. This team approach also promotes adherence by permitting maximum scheduling flexibility for families from Monday through Sunday, ensuring a spacing of two to three days between the twice-weekly sessions. The same two-PT team is responsible for provision of therapy to the child across both intervention phases, providing a constancy of rapport and interaction style between the PTs and child/parent across interventions. The advantages of this approach are likely to outweigh any intervention preference bias that might influence the actual treatment given. The consistency of treatment focus between PTs is enhanced through strict guidelines as to what treatment approaches may/may not be used, the menu-based and goal-directed intervention design, and the full documentation of all session components. These standardization strategies are very important in the PT intervention given the greater latitude for individual PT treatment variation.

A PTA is present for the Lokomat therapy intervention arm to assist with child set up to optimize the speed of set up, and help with Lokomat setting changes as well as documentation of setting changes throughout the session. The PTA is not present at the PT sessions unless the PT feels that one is required to ensure safety of the child in the walking-related activities. This additional use of the PTA is fully documented in the session log.

### Training of study intervention PTs and PTAs

#### Lokomat intervention

Certification for Lokomat use involves an extensive 2-day training session led by a Hocoma Lokomat licensed trainer. The first day of training familiarizes the PTs with the Lokomat and allowed practice with each other. At the second training day, PTs practice children with CP. Certification follows practice sessions with each other and with several other children with CP who were already receiving Lokomat therapy at a local clinic and hence not eligible for this study. This training approach ensures sufficient familiarity with the treatment progression protocol. Following approximately 3 months of practice, PTs attend a 1-day review session led by the same Hocoma licensed trainer. PTAs also attended the training sessions with the Hocoma trainer to learn the basic components of set-up of the child into the Lokomat orthosis. While they are not operating any of the robotic features of the Lokomat or altering any settings with the children, they do assist with the set-up and monitoring. Thus, it was felt that they needed to have a full understanding of the operation principles of the Lokomat.

#### Gait-related PT Intervention

The treatments involved in the gait-related PT intervention are a standard part of PT practice and the skills do not need to be taught. However, the process for selecting what is done from the study menu (specified within each of the GMFCS Levels) and treatment restrictions were covered in a 3-h orientation session led by the Co-PI (VW). PTAs are rarely involved in this arm, and when they are, it is to provide physical assistance. Thus, extra training is not required.

## Outcome measures

The primary outcome measures selected directly reflect those used in previous Lokomat studies (i.e., GMFM-66, 6MWT, Timed Up and Go and Canadian Occupational Performance Measure [COPM]) (Russell et al. 2000; Russell et al. 2013; Thompson et al. 2008; Williams et al. 2005; Law et al. 1990), and expand into other ICF areas including movement quality (Quality FM, basic gait assessment of spatiotemporal variables via electronic walkway and observational assessment) (Wright et al. 2008, 2014; Sorsdahl et al. 2008), ROM and spasticity (Tardieu) (Scholtes et al. 2006), advanced gross motor skills (*Challenge* Assessment) (Wright et al. 2012; Glazebrook and Wright 2014), functional abilities (i.e., PEDI Caregiver Assistance and ASK-30) (Haley et al. 1992; Young et al. 2000), and participation/QOL (i.e., step activity monitor, CAPE and KIDSCREEN) (King et al. 2007; Ravens-Sieberer et al. 2007; Bjornson et al. 2014). Collectively, these outcome measures provide comprehensive information in areas of activity and participation in alignment with the WHO ICF framework (World Health Organization 2001). Questionnaires are completed by the parent as well as the child if the child is 8 years or older. Each measure is used at each of the four assessment sessions. Personal motivation characteristics of the child (Dimensions of Mastery Questionnaire [DMQ]) (Miller et al. 2014) are assessed at baseline.

The COPM is completed by the child and parent with the assessor at the end of the assessment session, focusing on activity and participation goals. Since the child is not randomized to an intervention group until after the baseline assessment, these are goals that fit a gait-related functional mobility program generally and are not specific to the Lokomat or PT arms. This it is anticipated that these will be higher-level activity and participation related goals that are suitable to either intervention. Goal attainment scaling (GAS) (King et al. 2000) is done during the first two intervention sessions by the study PT with the child/parent. Working from the COPM goals that were created at baseline by the PT assessor, the study PTs re-script these into measurable GAS goals that fit with the intervention (PT or Lokomat) and what they learned about the child from review of their assessment results. This process allows the GAS aspect to be tied in directly to the perceived possibilities of the intervention arm, and fits with the specific outcome scaling process that is required within GAS, i.e., best suited to highly observable goal areas related to body structure and activity. Collectively it is hoped that the combined use of the COPM and GAS will provide a comprehensive evaluation of the extent and nature of goal accomplishments (GAS), as well as perceptions of performance abilities and satisfaction with abilities (COPM).

Within sessions, measures to monitor pain (Wong-Baker FACES^®^ Pain Rating Scale) (Wong and Baker 1988) and physiological effort (PCERT, heart rate) (Yelling et al. 2002) are obtained by the study PT/PTA. At the end of each treatment arm enjoyment is obtained by the RA using a modified version of the PACES (Moore et al. 2009) so that items evaluated make sense in the context of the Lokomat/PT sessions. A table of the measures (Table 1) provides details as to the GMFCS level each measure is linked with the format of administration (observational measure or questionnaire), time required and administration time points. Detailed information on each measure is provided in Additional file 4.

**Table 1:** Table of measures

Measure	GMFCS level	Type of measure/respondent^a^	Time to complete				
AT assessment				Baseline 1	Post-1	Baseline 2	Post-2
*Primary*							
GMFM-66	II & III	O/PT with child	30–45 min	x	x	x	x
6MWT	II and III	O/PT with child	10	x	x	x	x
*Secondary*							
GMFM STAND/WALK barefoot (GMFCS II and III) and with shoes/orthoses (GMFCS III)	II & III	O/PT with child	30	x	x	x	x
Timed Up and Go Test	II and III	O/PT with child	5	x	x	x	x
*Challenge *Assessment	II	O/PT with child	30	x	x	x	x
Quality FM	II and III	O–PT via GMFM video	30–60				
ROM/spasticity	II and III	O/PT with child	10	x	x	x	x
Gait evaluation: GAITRite^®^ walkway system; observational gait scale (video rating)	II and III	O/PT with child	15	x	x	x	x
PEDI-functional skills	II and III	Q/parent	15	x	x	x	x
ASK-30	II and III	Q/child or parent proxy	15	x	x	x	x
KIDSCREEN	II and III	Q/child or parent proxy	15	x	x	x	x
CAPE	II and III	Q/child or parent proxy	15	x	x	x	x
GAS and COPM	II and III	Q–child with parent	15	x	x	x	x
*Other*							
DMQ	II and III	Q/child or parent proxy	10	x			
Done at home							
Step-watch^®^ monitor	II and III	Child wears at home	5 days				
Done at sessions				Before session	Mid-point	End of session	Other time
Rating of exertion (PCERT)	II and III	Q/child	2		x	x	
Heart rate	II and III	O/child	1	x	x	x	
L-WALK (Lokomat session distance)	II and III	Lokomat system measure	N/A			x	
Pain scale (FACES)	II and III	Q/child	2	x		x	
PACES	II and III	Q/child	10				16th session
Motor learning strategy rating instrument (MLSRI)		O/rater scores video of intervention session	30				Session week 2 and 7

^a^Type of measure: O = PT observational assessment Q = interviewer introduced/guided questionnaire

### Training of the study assessors

The PT assessors have five to 20 years of experience in paediatric neurology and are familiar with the GMFM-66 from clinical practice. Regardless, a group training session will be done with the GMFM-66 (barefoot and shoes/AFO versions). All assessors independently score three GMFM videotapes (a child in each of GMFCS I, II and III) from the co-PI (VW)’s GMFM video-training bank (including a section on recognizing/resolving GMFM test administration errors common to the GMFM’s Stand and Walk dimensions). All must achieve a criterion score of 80% accuracy (the same level used with the original GMFM criterion test). The 6MWT, ROM, Tardieu Scale and child-/parent-report-questionnaires (DMQ, ASK-30, CAPE, KIDSCREEN, PEDI) have simple administration requirements and do not require further testing beyond the initial group training session. During assessments, the assessor also works with the study RA to administer the 15-min gait assessment (time distance parameters of gait) using our Gait-Rite system and gait video.

## Sample size

The study has been designed and powered to ensure adequate sample size for the primary research questions and analysis of children in GMFCS Levels II and III. The starting point for our sample size calculation consideration was a single group Lokomat study with a similar GMFCS sample and Lokomat use protocol in which the GMFM Stand and Walk mean scores changed by approximately 5 points (SD change = 7) (Borggraefe et al. 2010b). Our study is powered to let us detect a difference between the two interventions of three points (SD = 6: small effect size) on the GMFM-66, a magnitude of change that is considered to be a minimally clinically important difference (Oeffinger et al. 2008) when evaluating benefits of treatment. The reason for a difference change target in our study is that we are comparing two active interventions, each of which could result in positive change given the walking focus and boost for many of the children in therapy intensity. This sample size is also sufficient to handle the 6MWT comparisons and the small effect size observed by others in single group Lokomat studies in the 6MWT results (mean gains of 25 m [SD = 50 m], personal communication, Glenrose Hospital program, 2012).

For crossover designs, sample size calculations that link with a matched pairs *t* test calculation are appropriate (Senn 2002). A paired *t* test is also the underlying basis for the more sensitive repeated measures ANOVA that we have chosen to use so that we can include a period effect within the analysis. With an alpha of 0.025, beta of 0.20, and a detectable change score difference between intervention of thre points on the GMFM-66 (with an estimated SD of change of 6.0 points), a sample of 32 children is required (Hintze 2001). We increased the sample requirement to 40 to account for a potential drop out/protocol deviation rate of 20%. Sample calculation based on this generous drop-out rate will support the primary efficacy analysis that focuses only on the children who attend more than 70% of the sessions within one or the other intervention phase.

## Analyses

Data entry forms were built for each measure in the study to correspond to the paper forms that the assessors used. REDCap (Harris 2012) requires outer limits for each value, and each paper data sheet is double entered for purposes of data verification process. Collectively these features optimize the data entry accuracy. Study data will be kept for seven years following conclusion of the trial, at which point data will be destroyed following REB requirements.

Descriptive statistics and graphic displays will be presented for all outcomes for the PT and Lokomat interventions (pre, post-test and change scores). Equivalence between baseline_A_ and baseline_B_ scores will be evaluated for each outcome; a t-test or Wilcoxon test will be used in this comparison as appropriate. The primary analysis will be for the pre- to post-test differences (change scores) of the GMFM-66 and 6MWT between groups. Assuming normality of data, a repeated measures ANCOVA will be performed on GMFM-66 and 6MWT change scores to compare the effects of the Lokomat and gait-related PT interventions. The child’s relevant baseline score (baseline_A_/baseline_B_) will be used in the ANCOVA to increase analytic precision. The P value will be adjusted to 0.025 to handle the expected high correlation between the GMFM-66 and timed walk. Evaluation of an intervention order effect (i.e., being the first or second treatment), as well as the interactions with intervention group will be built into this analysis (Senn 2002). Secondary sub-analyses will be done in the same way within GMFCS II and III subgroups to determine any differential response for GMFCS Level.

Due to the lack of expectation of return to baseline in the break period, a second analytic approach will be taken as well using a generalized estimating equation (GEE) adjustment for repeated measures within subjects analysis (Baker et al. 2007). In this approach treatment effects are evaluated via regression analysis and the GEE method will allow the fit of two models. The first is for treatment effect, treatment-period effect, and differential carryover effect adjusting for the baseline scores, and the second is treatment effect and treatment-period effect with exclusion of any differential carryover effect. Factors hypothesized a priori to be associated with GMFM-66 change score magnitude will be entered within this model using a forward stepwise regression procedure, e.g., age, GMFCS Level, motivation (DMQ) score, ASK baseline score.

Finally, a third approach will be taken if there is significant evidence of a carryover effect and significant interaction effects. This will be to focus on the data from the first arm of the study, i.e., an ANCOVA adjusted for baseline GMFM-66 change scores of just the first intervention period (randomisation to either Lokomat or PT with 20 children per group). This comparison in itself would have a power of about 0.60 for the GMFM-66 (with alpha = 0.025 and a medium effect size of 0.50).

A similar set of analytic approaches will be used for the secondary outcome measures (GMFM Stand and Walk, Timed Up and Go, walk velocity/step and stride length [from GAITRite], observational gait scale, average daily step counts [step activity monitor data], Quality FM, *Challenge* Assessment, ASK-30, PEDI Part II, KIDSCREEN, COPM and GAS scores), using the baseline value of the measure of interest as a covariate within a MANCOVA procedure that will permit simultaneously handling of multiple secondary outcome measure change score evaluations. If both parent and child filled out the questionnaires (i.e., all children 8 years of age and up who were able to do so), the children’s data will be analysed as primary. Otherwise parent questionnaire data will be considered as primary. A comparison of child and parent questionnaire results will be done as a secondary analysis via an ANOVA in which both single point in time (baseline) and change scores will be evaluated. ROM and Tardieu scores will be summarized in a simpler manner, using descriptive statistics and graphical presentations to illustrate scores in each phase. The power of all primary and secondary ‘no difference’ analyses will be determined post hoc.

The primary analysis will include all children who attended ≥70% of sessions for each intervention, and those who discontinued one or both interventions for a reason directly related to the study (e.g., excessive fatigue associated with participation or issues related to dislike of one or both interventions), or dropped out of the trial due to an intervention-associated injury. In the case of dropout due to study fatigue or dislike of the intervention, a strong effort will be made to do a discharge assessment at the time of withdrawal. In the case of injury, it may be possible to follow the child and evaluate some of the outcomes when the child is able (post-minor injury). If the physical assessment is not possible due to compromised physical status, completion of the functional and participation measures may still be appropriate.

A second analysis will include all who adhered to ≥50% of each intervention as well as those who discontinued one or both interventions for reasons noted in the primary efficacy analysis. The third analysis will be an “intent to treat”, including the last available data from all participants regardless of level of adherence or reason for withdrawal. As outlined above, a strong effort will be made to do a discharge assessment at time of withdrawal. Demographic characteristics, baseline scores and change scores of these three adherence groups will be evaluated to determine any systematic differences in their characteristics or outcomes.

Data from the session summary sheets will be compiled. Treatment categories, Lokomat parameter adjustments, and session activities will be summarized through graphic and tabular summaries, and related descriptive information from therapists’ comments will be reported.

## Adverse events (AE)

At the start, midpoint and end of each session heart rate is taken via pulse, and children are asked about any pain/musculoskeletal issues using a visual analogue scale with pain ratings done as per the FACES^®^ scale (Wong and Baker 1988) and a generic body diagram. All areas of the child’s skin that underlie the Lokomat straps and cuffs are checked by the study PT before and after every Lokomat session, with any areas of redness or skin breakdown marked on the body diagram. The expectation is that any areas of redness that occur during Lokomat therapy are transient and fully resolved by the next session. Failure to resolve between sessions requires adjustment of the straps or padding, realignment of the Lokomat set-up, use of skin protection tape, or withholding the next session as appropriate. At the end of each session, the study PT enters all AE information noted at that session on an AE summary sheet and alerts the study RA to review the sheet. The RA and PI classify the event in terms of severity and attribution to the Lokomat or PT session, and determine necessary follow-up action according to a pre-set action protocol approved by the REB. This process ensures that a prompt response, precautions and reporting to the REB can be taken as required. All occurrences of open wounds or fracture are reported by the co-PIs within 24 h of the session to the REB with treatment offered through Holland Bloorview. AEs are monitored by a Safety Monitoring Committee (SMC) acting independently of the investigators and study funders. This SMC consists of a pediatrician, methodologist/researcher, nurse, pediatric orthopaedic surgeon, PT, and a lay person. The SMC meets every four months to review any AE forms that have been filed since time of the previous meeting, or convenes a special interim meeting to review any event that required reporting to the REB. Early stopping of the trial will occur if a recommendation to terminate the study is made by the SMC by majority vote. This may occur at any time, with decisions reported to the PI and REB by the SMC Chair immediately by telephone and email along with the reason for their decision. Withdrawals for reasons other than safety concerns will not be cause for study termination.

## Discussion

This study’s comparison of the Lokomat intervention with a gait-related PT program is the first randomized trial that we are aware of in paediatric CP that incorporates a broad set of outcomes spanning the body structure/function, activity and participation aspects of the ICF-CY (World Health Organization 2007) as well as health-related quality of life (McDougall et al. 2010). To address co-intervention limitations in previous trials, RAGT is being delivered in isolation of other therapies. Group comparisons will be strengthened by having comparable group size, using a therapy intensity and duration designed to promote functional change with time for learning and transfer into daily life. The inclusion of individualized goals and use of outcomes reflective of real-life activity and participation are unique to this study. The detailed reporting of intervention protocols and individual goals will permit operationalization of therapy, extending potential benefits beyond the study cohort and informing transfer of the intervention approach into clinical practice. The intervention session design is strengthened by the input and experience of participating PTs from the CDP clinical area.

The evaluation of an order effect (Lokomat or PT first) may provide important insights into clinical integration of Lokomat therapy with gait-related PT. For example, if overall gross motor outcomes are better when Lokomat therapy is given before a block of PT (e.g., quality gains on the Lokomat can be used to set the stage for functional and participation gains with PT), this may be of value when determining the sequencing of blocks of therapy to achieve gait-related goals.

If efficacious, RAGT holds promise for transforming treatment for children with CP and other neurological disorders by potentially making walking therapy more stimulating and engaging as well as more inclusive of a range of children. We expect our results to inform further research with children with other neurological disorders such as acquired brain injury and spinal cord injury, in addition to a multi-centre factorial design study with children with CP.

The findings of the trial will be disseminated through peer-reviewed journals, national and international conferences. Strategies to integrate motor learning principles into practice will be communicated to physiotherapists through in-person workshops, informed by analysis of session content using the MLSRI (Kamath et al. 2012). Upon trial completion, a summary of findings will be communicated to participants and families. Intervention protocols will be reported in detail, with results reported in accordance with CONSORT guidelines.

## Additional files

**Additional file 1.** Summary of Lokomat Research with Children with Cerebral Palsy.

**Additional file 2.** Lokomat Training Protocol. A detailed outline on content and reporting procedures for Lokomat treatment arm.

**Additional file 3.** Physiotherapy Protocol. A detailed outline on content and reporting procedures for physiotherapy treatment arm.

**Additional file 4.** Description of Measures used in the Trial. Details on outcome measures used in the trial including purpose and description of measure, rationale for use in trial, and summary of psychometric properties.
