# Supplementary material for: Comparison of a robotic-assisted gait training program with a program of functional gait training for children with cerebral palsy: design and methods of a two group randomized controlled cross-over trial
Source: Springerplus. 2016 Oct 28;5(1):1886. doi: 10.1186/s40064-016-3535-0 (PMC5084143; doi:10.1186/s40064-016-3535-0)
Supplement: Supplementary file 1 — Additional file 1. Summary of Lokomat Research with Children with Cerebral Palsy. [file 40064_2016_3535_MOESM1_ESM.pdf]

Additional file 1
Summary of Lokomat Research with Children with Cerebral Palsy: Multi-session Intervention Studies

(Listed in chronological order of publication)

Study	Study design and sample characteristics	Intervention Details and Main Outcome Measures	Results and Conclusions	Limitations
Meyer-Heim et al. 2007	**Design**: Single group, prospective.<br>**Sample**: 26 children, ages 5 to 19 years, with central gait impairment (19 children had CP, Gross Motor Function Classification System [GMFCS] Levels I to IV). Mix of in-patients and out-patients. The in-patients also received a standard program of physiotherapy (PT).	Two to five, 25- to 45-minute Lokomat (LOK) sessions per week for total of 10 to 20 sessions (10-13 for outpatients). Support and gait speed progressed as tolerated.<br>**Measured** using six minute walk test (6MWT) – walking endurance, 10 metre walk test (10mWT) – walking speed, Gross Motor Function Measure (GMFM-88) Stand and Walk Dimensions pre- and post-intervention.	Both gait speed and walking endurance increased significantly (P<0.01) following the LOK program. Also significant gains in GMFM Stand and Walk scores (P<0.05).<br>**Concluded** that LOK is a promising intervention that allows higher intensity gait therapy than with usual PT approaches. The use of the LOK was considered to be successfully and safely integrated into the in-patient PT program.	Since this was a single group study (not an RCT), were not able to claim cause and effect, only association (e.g., children might have improved just due to the PT that the in-patients also received or to increased therapy hours in general). Limited scope of outcome measures (gait and gross motor function only). Regardless, this is a foundational first published study for use of the LOK with children with CP.
Borggraefe et al. 2008	**Design**: Case report.<br><br>**Sample**: 6-year old boy with bilateral spastic CP, GFMCS Level III.	Twelve LOK sessions (mean duration of 34 minutes) over course of three weeks. Velocity progressed from 1.1km/h to 1.8km/h; body-weight support (BWS) reduced from 50% to almost 0%; guidance force stable at 50%. The child was able to watch his favourite DVD for motivation.<br>**Measured** using 10mWT and 6MWT and GMFM-88, pre-, post-intervention and 4-months follow-up.	Score for GMFM Stand doubled, and Walk dimension scores increased as well. Walking speed and endurance both increased and were preserved at the 4-month post follow-up period.<br>**Concluded** that use of LOK with children with central gait impairment might help them to gain motor function.	Case-report with only one child. No single subject design trend analyses were performed.<br>Child had a botulinum toxin-A injection two months prior, which may have been a confounder for outcomes (noted by authors). Use of DVD movie as distraction may have limited extent of use of motor learning strategies in the sessions. Authors suggested that the LOK biofeedback system needs to be better adapted to younger children to promote use and support active learning in the sessions.

Study	Study design and sample characteristics	Intervention Details and Main Outcome Measures	Results and Conclusions	Limitations
Meyer-Heim et al. 2009	**Design**: Single group, prospective.<br><br>**Sample:** 22 children age 4.6–11.7 years with CP, GMFCS Level II-IV.	Three to five LOK sessions, 45–60 minutes/week over a three to five week period. Support and gait speed progressed as tolerated. **Measured** using 6MWT, 10mWT, GMFM-88 Stand and Walk Dimensions pre- and post intervention.	Both maximum gait speed and GMFM Stand scores increased significantly post LOK (P<0.01) Observed mean score gains in walking endurance and GMFM Walk scores were not significant (minimum P>0.05).<br>**Concluded** that children with CP could benefit from LOK training as far as improving functional gait parameters.	Since this was a single group study (not an RCT), were not able to claim cause and effect, only association (e.g., children might have improved just due to the PT that the in-patients also received or to increased therapy hours in general). Limited scope of outcome measures (gait and gross motor function only). Not clear from the paper if the sample is distinct from that of their 2007 study or there was overlap with some of its participants with CP under age 12.
Borggraefe et al. 2010a	**Design**: Single group, prospective.<br><br>**Sample**: 14 children, ages 4.5 to 19 years, bilateral spastic CP, GMFCS I to IV.	Twelve, up to 50-minute LOK sessions given over three weeks. Velocity progressed from 1.1km/h to 1.8km/h; BWS started at 100% and was reduced as tolerated to limit of start of collapse into knee flexion. Guidance force (impedance control) was reduced from 100% until too much child activity caused LOK to stop, then increased by 5%. No use of biofeedback. Allowed to watch favourite DVD if attention waning. **Measured** using 6MWT (endurance), 10mWT (speed), GMFM-88 Stand and Walk at pre-, post-intervention and 6-month follow-up.	Significant increase in GMFM Stand (P =0.008) and Walk abilities (P=0.01) from baseline to post-treatment assessment and continuing into 6-month follow-up (P<0.05). Increased endurance (6MWT) from baseline to post-treatment assessment (P=0.03) gait speed (P=0.006) Improvements in motor function were maintained compared to baseline through the 6-months follow-up (only gait endurance analysis was not significant, P = 0.10).<br>**Concluded** that there appears to be potential for retention of gains in the long term.	Since this was a single group study (not an RCT), were not able to claim cause and effect, only association. The single group studied was a heterogeneous group as far as CP type and GMFCS Level, and no group sub-analyses done. The training period chosen was very short and intense and while in line with motor learning principles, may be unrealistic for clinical use. Limited scope of outcome measures (gait and gross motor function only). There was a lack of a control group, and the therapy during the 6 month follow-up period was not standardized nor monitored, i.e., seven children actually received more LOK therapy.

Study	Study design and sample characteristics	Intervention Details and Main Outcome Measures	Results and Conclusions	Limitations
Borggraefe et al. 2010c	**Design**: Single group, prospective.<br><br>**Sample**: 89 children, mean age 11.4 (SD 4.5) years. Fifty-eight with CP. Other diagnoses: paraplegia, brain injury, other neurologic.	Twelve, up to 50-minute LOK sessions given over three weeks. Velocity progressed from 1.1km/h to 1.8km/h; BWS started at 100% and was reduced as tolerated to limit of start of collapse into knee flexion. Guidance force (impedance control) was adapted according to PT's judgment. Worked hard to promote full engagement for learning, but allowed to watch favourite DVD as needed to sustain compliance. **Measured** using GMFM-88 Stand and Walk at baseline and at treatment completion, as well as walk distance and time covered in LOK sessions.	Significant improvement of GMFM Stand (P=0.001), and GMFM Walk scores (P<0.001). Significantly greater mean total distance (P<0.001) and total time (P=0.002) walked in LOK were observed in patients with less impairments (GMFCS I and II). **Concluded** that the gains in standing abilities as well as walking point to the added effect of LOK on postural stability. Unclear whether greater gains in those in GMFCS I and II was due to greater ability to train for longer or to higher ability to make gains in motor function than those in GMFS III and IV.	Since this was a single group study (not an RCT), were not able to claim cause and effect, only association. Appears to be an extension of the Borggraeffe et al. 2010a study to permit a look at GMFCS Level response rather than extended response. The training period chosen was very short and intense and while in line with motor learning principles, may be unrealistic for clinical use. Limitation of scope of outcome measures (gross motor function only) with walking distance measured only within sessions not as 6MWT.
Borggraefe et al. 2010b	**Design**: Single group, prospective.<br><br>**Sample**: 89 children, ages 4.5 to 20 years, bilateral spastic CP, GMFCS I to IV.	Safety data from all patients participating in a trial of LOK therapy at two centres were tracked and analysed. Sessions consisted of 12 sessions (outpatient) or 15 to 20 (inpatient) of maximum 50-minute LOK sessions given over a three week period. Body weight usually started at 50% and adjusted to optimize gait pattern. Guidance force was individualized as tolerated.	Total of 47 adverse events in 38 of 89 children. Most common were muscle pain (n=16), joint pain (14), skin erythema (12), open skin (4) and tendinopathy (1). In five children, LOK had to be put on hold. There were no serious adverse events. There were no associations with age, number of sessions, total distance walked or duration of therapy. **Concluded** that with careful fitting to the child's body structure and function at each session, the LOK is a safe intervention for children with central gait impairments.	As noted by the authors, the classification of adverse events into mild, moderate and severe was decided arbitrarily by the investigators based on their centres' experiences. Both centres were experienced in use of LOK – data did not take into account the learning curve that might happen with children early in a centres' LOK use.

STUDY	STUDY DESIGN AND SAMPLE CHARACTERISTICS	INTERVENTION DETAILS AND MAIN OUTCOME MEASURES	RESULTS AND CONCLUSIONS	LIMITATIONS
Drużbicki et al. 2010	**Design**: Two group, randomized controlled trial.<br><br>**Sample**: 18 children and youth, 6-14 years with spastic diplegic CP, GMFCS Level II or III.	Experimental group (n=9), who received 20, 45-minute LOK sessions plus physiotherapy (PT) over four weeks. LOK settings decided by physiotherapist. LOK feedback screen used within sessions.<br>Control group (n=9) who received 20, 45-minute PT sessions over four weeks.<br>**Measured** balance with eyes open and eyes closed by means of a stabilometric Zebris platform with force sensors used to calculate static and dynamic forces. Assessor was blinded to treatment group.	The experimental group showed significant balance gains with LOK treatment as measured by confidence ellipse width (P=0.06), and vertical deviation (P=0.038), when tested with eyes open. With eyes closed, significant declines in measurements were observed with confidence ellipse width (P=0.028), confidence ellipse area (P=0.015), total track length (P=0.007), and vertical deviation (P=0.02). No significant changes noted in control group.<br>**Concluded** that the LOK may have beneficial impact on improving balance in children with CP.	More homogenous in sample characteristics than previous trials but limitations include small sample size and drop-out of 4 children (50%) in the control group. Analysis did not directly compare changes between groups. The PT program details were provided only in brief (focus on motor control, improved stability in sit and stand and walking). Outcome scope was limited: the Zebris platform was the only measure used, and thus results cannot be generalized to enhancing walking abilities or motor function.

Study	Study design and sample characteristics	Intervention Details and Main Outcome Measures	Results and Conclusions	Limitations
Romei et al. 2012	**Design**: Two group, non-randomized clinical trial<br><br>**Sample**: 19 children, ages 4-16 years, with spastic bilateral CP, GMFCS Levels I to III.	LOK + task-oriented physiotherapy (TOP) group received 20, 30-minute LOK sessions over period of 10 weeks (LOK 2x/wk + PT 2x/wk) followed by 20 30-minute TOP sessions over period of 10 weeks. In the LOK, BWS was fixed at 50%, and guidance force fixed at 100%. Speed was increased (by 10% every five sessions [young group], 20% every five sessions [older group]), but not altered within sessions. The TOP was a standardized PT program of gait, balance and strength.<br>Intensive TOP (ITOP) group received 40, 30-minute sessions over period of 10 weeks (PT 4x/wk). TOP was a standardized PT program of gait, balance and strength (same as LOK children received).<br>**Measured** using GMFM-66 and Stand and Walk Dimensions (hoes and devices permitted), 6MWT and 3D gait analysis (Gillette Gait Index [GGI], and Functional Assessment Questionnaire (FAQ) at pre-, post-intervention and 3-month follow-up.	Both LOK+TOP and ITOP groups showed significant improvements of GMFM-66, Stand and Walk ($P < 0.05$) but there were no differences between groups (P value not given). 6MWT, GGI and FAQ did not significantly change in either group (P value not given).<br>**Concluded** that LOK+TOP was as effective as ITOP alone, and LOK considered safe and feasible for clinical use in rehabilitation programs for children with CP	The sample was not allocated to treatment group randomly (i.e., the first 9 children recruited got into LOK+TOP and the next 10 into ITOP). Although there were no statistically significant differences between groups, there were notable mean score differences at baseline such as age, height and weight. Separate analyses not presented for the post-intervention and 3 month follow-up results (just mean score results tables). Small sample size with possibility of Type II error for no difference results. While there were no drop-outs in either group, treatment adherence was not reported. Limited scope of outcome measures (gait and gross motor function only).<br>The lack of individualization of the BW and guidance support may have limited motor learning possibilities and other progress.<br>TOP content was not provided so no clinical transferability possible. No indication of blinding of assessors to group.<br>While conclusions indicated that LOK can increase children's motivation, objective data not provided to support this.

Study	Study design and sample characteristics	Intervention Details and Main Outcome Measures	Results and Conclusions	Limitations
Pajaro-Blazquez et al. 2013	**Design**: Single group, retrospective.<br><br>**Sample**: 51 children, mean age = 10±4, with CP (spastic CP, n=44) who had previously undergone LOK training sessions. GMFCS Levels I to IV.	Of the 69 interventions session sets provided, 34 participants completed 1 Lokomat session set, 17 participants completed 2 Lokomat session sets, and one participant completed 3 Lokomat session sets. The mean number of sessions done within a set was 11 (range of 6 to 19 sessions). The mean session duration was 26 minutes.<br>**Measured** using 6MWT and 10mWT at pre- and post-intervention.	There was significant difference in walking endurance (6MWT $P<0.01$) and walking speed (10mWT, $P<0.01$) after LOK training.<br>**Concluded** that LOK training may be a therapeutic option for improving gait speed and endurance in children with CP.	Despite the benefits of a larger sample size than previous studies, there are validity issues associated with retrospective study designs. Single group design also means inability to attribute cause and effect, just association of change with intervention. Large variability in the session protocols (frequency, intensity). Limited scope of outcome measures (just walking endurance and time). Missing data for both walk tests, e.g., 34 interventions did not have pre- and post 6MWT data. Paired data (e.g., 17 children had their first and second LOK session set results counted in the pre and post-analysis) reduces internal validity of the results.
Druzbicki et al. 2013	**Design**: Two group, randomized controlled trial<br><br>**Sample**: 52 children ages 6 to 13 years, with spastic diplegic CP, GMFCS II-III.	Study group: 20, 45–minute LOK sessions over 4 weeks with active verbal cueing by PT and visual graphic representation of walking pattern shown on monitor. BW and velocity were adjusted as tolerated by the therapist in the LOK from session to session. The biofeedback screen from the LOK was used. Also had an exercise program aimed at increasing balance, motor control and walking skills (time and frequency unclear).<br>Control: 20, 45-minute PT sessions aimed at increasing balance, motor control and walking skills (same as that done in the LOK group).<br>**Measured** using temporospatial and kinematic gait analysis via a BTS Smart motion analysis system performed pre- and post-intervention. Blinding of assessor and data analysts to group.	An increase in mean gait speed was noted in both groups following treatment however this was not statistically significant ($P=0.59$). There were no statistically significant changes in gait parameters in the two groups other than hip flexion range in both groups ($P=0.006$).<br>**Concluded** that the limitations of the study reduce strength of conclusions about lack of effectiveness and that further research is needed.	This study should be regarded as preliminary only, and further studies are needed to investigate the impact of the LOK treatment on children with CP. In the LOK group, it is not clear how the PT exercise program factored into the 45-minute time indicated for LOK therapy. The total length of time (weekly frequency) over which the 20 LOK or PT group sessions were given is also not clear. Seventeen participants dropped out of control group and do not appear to have been considered in part of the analysis (not included in baseline equivalence table). Limited scope of outcome measures (focused only on gait analysis).

Study	Study design and sample characteristics	Intervention Details and Main Outcome Measures	Results and Conclusions	Limitations
Schroeder et al. 2014a	**Design:** Single group, four assessment time points: pre-baseline (V1), baseline (V2), post-intervention (V3), 8-week follow-up (V4).<br><br>**Sample:** 18 children/adult ages 5.0 to 21.8 years (mean 11.5), with bilateral spastic CP, GMFCS Levels I-III.	Participants completed 12 sessions of 30-60min RAGT training over three weeks. Walking duration set as tolerated by patient, termination of session when physical exhaustion evident. Body weight support decreased from 100% to point before knee collapse in flexion during stance phase. Guidance force adjusted as per clinical judgment. Verbal feedback provided by therapist.<br>**Measured** using GMFM 66, self-selected and maximum walking speed, COPM.	GMFM 66 (total score p=0.001; Dimension D p=0.004; Dimension E p=0.005) and COPM (Performance p=0.01; Satisfaction p=0.049) showed significant improvements at V3 and V4 as compared to V1 and V2. Change in gait speed (maximal and self-selected) not significant. Mean gains of 2.1 points (met the change target for clinically important) in COPM performance and 1.8 points in COPM goal satisfaction. Age, GMFCS level, and past RAGT experience correlated negatively with GMFM improvement. No correlation with these variables and COPM improvement.<br>**Concluded** that following RAGT training, significant changes in activity and participation domain of ICF are observed.	Since this was a single group study (not an RCT), were not able to claim cause and effect, only association. Wide age range of participants, many had experienced RAGT training. COPM goals not described therefore difficult to interpret. Active participation of participants said to be achieved via variations in speed, body weight support and guidance force.
Schroeder et al. 2014b	**Design:** Single group.<br><br>**Sample**: 83 children, ages 4 and 18 years, GMFCS Levels I-V, with bilateral spastic CP (n=69), unilateral CP (n=3), ataxic CP (n=3), hereditary spastic paraparesis (n=6), and genetic syndrome including spasticity (n=2).	Participants completed 12 sessions of LOK training over 3 weeks of 30-60min as per participants' tolerance. Body weight support and guidance force were reduced over course of intervention. Therapists provided verbal motivation to promote active participation of child.<br>**Measured** using pre/post GMFM-66 total score, GMFM-D score (standing abilities), and GMFM-E score (walking, running, and jumping abilities). Effect of baseline GMFM-66 score, age, aetiology, sex, and use of botulinum toxin therapy analyzed.	Large effect size change in GMFM-66, GMFM-D and GMFM-E with 2.5. to 5.5 point improvement. Significant linear association (P < 0.01) between GMFM-66 baseline score and both GMFM-66 and GMFM-E scores. GMFM-D improvement was inversely associated with age. Hyperbolic pattern of improvement related to GMFM-66 baseline score with greatest change at 65% (i.e., score range for child in GMFCS Level II) .No other determinants significant.<br>**Concluded** that age and GMFCS Level are significant predictors of GMFM outcomes.	Since this was a single group study (not an RCT), were not able to claim cause and effect, only association. Only GMFM assessed, which does not capture change in domains beyond gross motor ability. Unbalanced sample for patient specific determinants (majority of participants GMFCS Level III, bilateral spastic CP). GMFCS Level not analysed as a determinant, rather inferred from GMFM score. GMFCS Level is a more clinically usable determinant for prescription of LOK training.

References

Borggraefe I, Kiwull L, Schaefer JS, et al (2010a) Sustainability of motor performance after robotic-assisted treadmill therapy in children: an open, non-randomized baseline-treatment study. Eur J Phys Rehabil Med 46:125–131.

Borggraefe I, Klaiber M, Schuler T, et al (2010b) Safety of robotic-assisted treadmill therapy in children and adolescents with gait impairment: a bi-centre survey. Dev Neurorehabil 13:114–119.

Borggraefe I, Meyer-Heim A, Kumar A, et al (2008) Improved gait parameters after robotic-assisted locomotor treadmill therapy in a 6-year-old child with cerebral palsy. Movement Disorders 23:280–283.

Borggraefe I, Schaefer JS, Klaiber M, et al (2010c) Robotic-assisted treadmill therapy improves walking and standing performance in children and adolescents with cerebral palsy. Eur J Paediatr Neurol 14:496–502.

Druzbicki M, Rusek W, Snela S, et al (2013) Functional effects of robotic-assisted locomotor treadmill thearapy in children with cerebral palsy. J Rehabil Med 45:358–363.

Drużbicki M, Rusek W, Szczepanik M, et al (2010) Assessment of the impact of orthotic gait training on balance in children with cerebral palsy. Acta Bioeng Biomech 12:53–58.

Meyer-Heim A, Ammann-Reiffer C, Schmartz A, et al (2009) Improvement of walking abilities after robotic-assisted locomotion training in children with cerebral palsy. Arch Dis Child 94:615–620.

Meyer-Heim A, Borggraefe I, Ammann-Reiffer C, et al (2007) Feasibility of robotic-assisted locomotor training in children with central gait impairment. Dev Med Child Neurol 49:900–906.

Pajaro-Blazquez M, Shetye R, Gallegos-Salazar J, Bonato P (2013) Robotic-Assisted Gait Training in Children with Cerebral Palsy in Clinical Practice. Converging Clinical and Engineering Research on Neurorehabilitation Biosystems Biorobotics 1:29–33.

Romei M, Montinaro A, Piccinini L, et al (2012) Efficacy of robotic-assisted gait training compared with intensive task-oriented physiotherapy for children with Cerebral Palsy. The Fourth IEEE RAS/EMBS International Conference on Biomedical Robotics and Biomechatronics 1890–1894.

Schroeder AS, Homburg M, Warken B, et al (2014a) Prospective controlled cohort study to evaluate changes of function, activity and participation in patients with bilateral spastic cerebral palsy after Robot-enhanced repetitive treadmill therapy. Eur J Paediatr Neurol 18:502–510.

Schroeder AS, Kries Von R, Riedel C, et al (2014b) Patient-specific determinants of responsiveness to robot-enhanced treadmill therapy in children and adolescents with cerebral palsy. Dev Med Child Neurol 56:1172–1179.
