# Supplementary material for: Comparison of a robotic-assisted gait training program with a program of functional gait training for children with cerebral palsy: design and methods of a two group randomized controlled cross-over trial
Source: Springerplus. 2016 Oct 28;5(1):1886. doi: 10.1186/s40064-016-3535-0 (PMC5084143; doi:10.1186/s40064-016-3535-0)
Supplement: Supplementary file 2 — Additional file 2. Lokomat Training Protocol. A detailed outline on content and reporting procedures for Lokomat treatment arm. [file 40064_2016_3535_MOESM2_ESM.pdf]

Holland Bloorview
Kids Rehabilitation Hospital

Lokomat Training Protocol

FORMAT OF SESSION

The goal is **30 minutes of active treatment** on the Lokomat (starting at 15 minutes if necessary and progressing time as quickly as possible with each successive session) plus 2-5 minutes of overground walking practice before the session and 5 minutes of overground walking practice post-session. * *Please do not progress beyond 30 minutes of active training in the Lokomat for any child during this research trial.* Each Lokomat session will include 10 introductory minutes of conversation, completion of pain form and heart rate, goal review and planning at the start of the session and 5 minutes at the end of the session to wrap-up. Including 5-10 minutes of set-up time in the Lokomat, the total duration will be a maximum of 65 minutes.

UNDERLYING TRAINING PRINCIPLES

1. **Amount of weight support, guidance force and treadmill speed** should be set and **re-adjusted** during the sessions to maximize the child's active participation. Continually think about and plan how to systematically alter Lokomat settings to work on the child's gait goals and provide **progressively increasing challenge and gait variety**. Use the session tracking graphs to indicate every change that you make to the settings, and use the Notes section to indicate why you chose to make changes. While the Lokomat captures this information, tracking it yourself will heighten your awareness of parameter changes.

2. Try to keep child **focused on active tasks** (walking work or integrated upper body activities) at all times. Avoid use of videos as a distraction from walking. These may be used during set-up but should be off during the session.

3. **Maximize use of feedback** through different approaches including **verbal, visual (mirror and modelling),** and use of the **Lokomat biofeedback screen**. Fade feedback as required to increase the challenge and to promote the child's reliance on intrinsic feedback. Draw the child's attention to **intrinsic feedback** by asking them to describe the feelings in his/her legs, or cueing the child to focus on specific sensations.

4. **Increase motivation** by using a whiteboard recording approach to track the child's accomplishments during the session, e.g., count of number of times child achieves hip flexion target. Set small goals to increase the child's motivation and reward him/her with stickers if favourable for motivation outcomes. The session plan can also be displayed on the whiteboard to keep the child focused and motivated on each task.

5. Child should **only be passively walking** in the Lokomat when he/she needs a brief 2- or 3-minute 'free ride' rest (full or almost full support and guidance). No more than 20% of the total session time in the Lokomat should be a 'free ride'. More free ride time than this is indicative of child fatigue or reduced engagement, and means that you should probably end the session. Make sure all 'free rides' are recorded on the log sheet as you go along in the session.

LOKOMAT TRAINING

Training is to be conducted as outlined in Chapter 6 of the Hocoma Lokomat manual (Hocoma AG, Switzerland, www.hocoma.com, pp.27 to 43) with a focus on use of active motor learning principles. The usual session format in the Lokomat is:

- **Set-up:** There will be about 5 to 10 minutes set-up of child in the Lokomat. A brief passive stretch can be done, if needed, prior to putting the child in the Lokomat (parent or therapist can do this). Please record in the log if this time is added to any of the sessions. **The 30-minute timing for the Lokomat session starts after the child's walking has been properly synchronized to the treadmill speed and the desired starting gait pattern/settings have been achieved.**

- **Begin with Lokomat settings based on recommendations from previous session** (range of motion, treadmill speed, weight support, guidance force). For the first sessions, the starting weight support should be in the 50 - 60% range, while guidance force starts at or close to 100% and is then reduced as tolerated (Lokomat manual, chapter 6). When the child is settled, work on gait goals using verbal and/or manual guidance and exercise activities (e.g., ideas in the Polish manual). Implement the Lokomat's biofeedback features as suitable to the child's abilities/goals. At each session, refer back to the child's goal sheet as a reminder of the motor abilities, gait features and activities to work on.
- **Adjust Lokomat parameters** at various points during the session: reduce support, increase speed, and/or alter guidance force. Make these alterations singly or in combination as appropriate to provide specific training for the child's gait goals. Remember that changes can be made to one leg and not the other, if desired. When possible, **take the child to the edge of support and guidance settings at least once** (separately or simultaneously as appropriate) in the session to maximize the challenge. Taking the child to the edge may mean gradually moving them into the zone where the Lokomat stops because child cannot manage the gait. At this point reset the support/guidance force to be just inside the setting where the gait was no longer sustainable and work at that level until child shows signs of tiring, discomfort or disengagement. Back off as needed to an easier setting, i.e., the setting used at session start. See sample of how to record these changes on the last page. There is early evidence that getting individuals to **take as many steps as possible** in a session leads to greater gains in walking abilities. This means trying to get the treadmill speed as high as possible for at least short bursts of time.
- Try to incorporate a **period of dual task activities** while walking, such as upper body tasks and games, overhead reaching, arm swinging (add poles/hand weights), and cognitive activities (counting, singing). Move one or both hand rails to the side as tolerated when doing upper body work. Make sure the child can still reach the safety stop by repositioning the button if handrails are removed.
- **Set a total walking distance target** for each session. Mark the distance achieved at the end of each session on the session log.

OVERGROUND WALKING PRACTICE

There will be 2 to 5 minutes of overground walking with shoes/AFOs at the **start *and* end** of every session to encourage assimilation of Lokomat session work to regular walking. This is an important chance for motor learning, as the child will be focused on correcting targeted gait deviations based on the feel of how it was done in the Lokomat. Ask the child to tell you and then show you what they worked on in the Lokomat (e.g., big steps, standing tall, knee straight). If they can't remember at the beginning of the session, review the key elements with them (information from the last sessions will be noted in the log sheet and session summary from previous session).

HOMEWORK

While we do not want you to give the children any physiotherapy exercises to work on during this arm of the trial, you can encourage them to practice the elements of gait they focused on during overground walking at the end of the session. Suggest they practice at home each day when they walk, and help them to select an environment in which to practice (i.e. hallway at home). Additionally, the children can be encouraged to do 'mind gym' work when they spend time thinking about how to walk like they do in the Lokomat.

* All children will have had two Lokomat acclimatization visits before the series of 16 intervention sessions begins. These visits are designed to introduce the child to the feel of the Lokomat; to ensure that we determine a good fit and suitable settings to start with respect to guidance force and weight support; to explain its principles to the child and parent; and lastly, to let the child become familiar with the exertion and pain rating forms. In the first session, the focus is on fit and air walking with no more than 5 minutes of treadmill walking. In the second session, the period of treadmill walking is progressed to 15 minutes, as tolerated, as we work hard to find the settings that will optimize the child's gait pattern. Assuming all is well at this point, the next session is the official intervention session 1. If further set-up adjustments are needed, a third acclimatization session is provided. If acceptable fit and gait are not achieved by the end of this third session, the child will be withdrawn from the trial. This situation is not expected given the rigorous screening criteria for the study prior to study enrolment.

Holland Bloorview
Kids Rehabilitation Hospital

SAMPLE

Lokomat Session Log

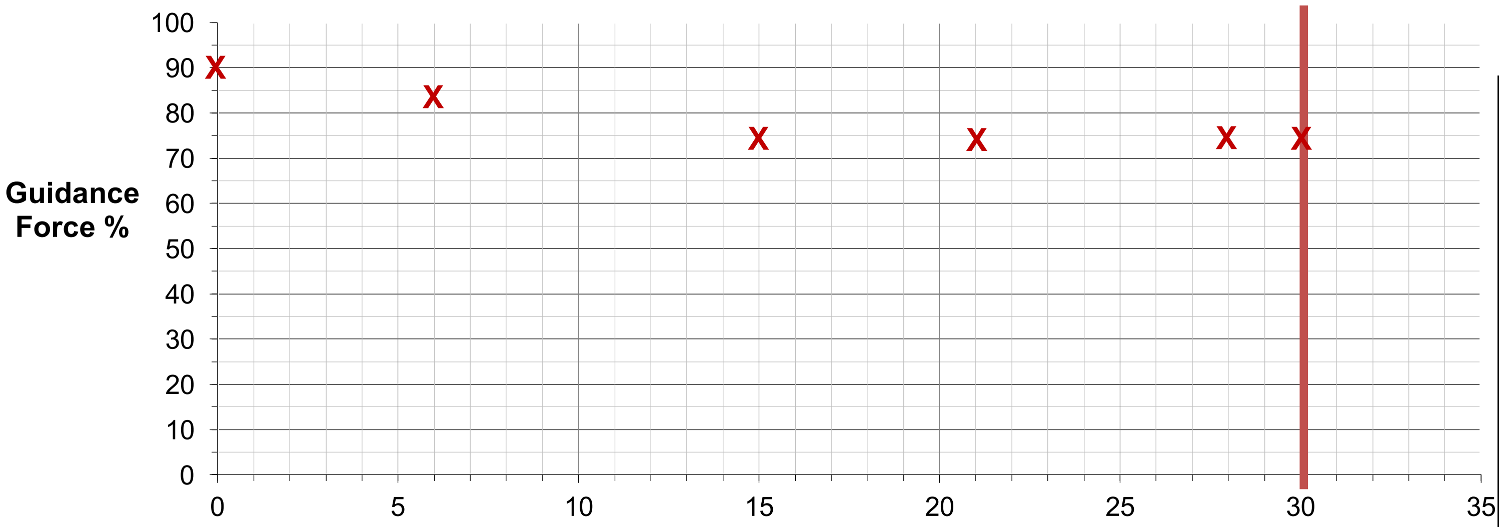

Remarks on Guidance Force:

Fill in details of why guidance force was adjusted.
(i.e.) Started at 90% as per previous session. Guidance force reduced to 85% to step over objects. Maintained as client tolerated reduced guidance. Guidance force was then decreased to 75% to increase challenge.

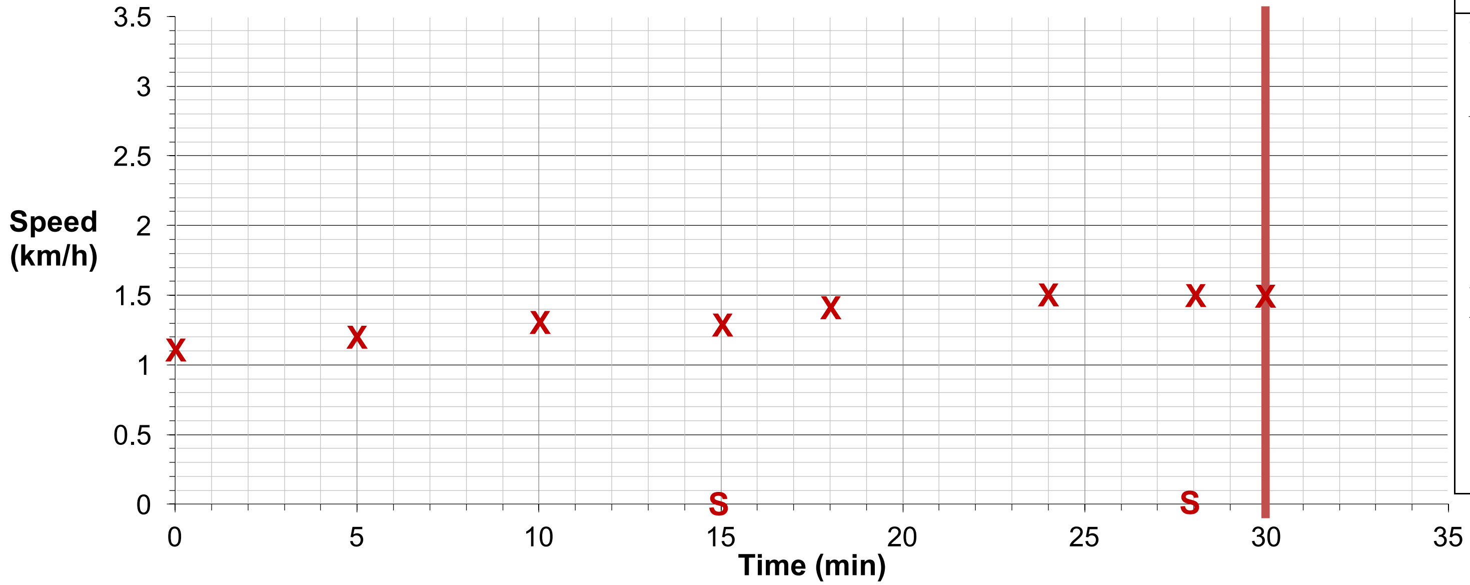

Remarks on Speed:

Fill in details of why speed was adjusted.
Speed incrementally increased during session as tolerated. Focus of session was to increase speed to 1.5 km/h with a guidance force below 80%.

Don't Forget to mark an X at child's start level (Y axis) for support, guidance and speed. When any of these are changed, mark the new level with an X. Whenever the Lokomat has to be briefly stopped (for any reason), mark S (stop) on bottom time axis of speed graph on this page. Mark a vertical line down all graphs to indicate end of session.

Hilderley, Fehlings, Lee, Wright. Holland Bloorview Kids Rehabilitation Hospital, Toronto, Canada, 2016

Holland Bloorview
Kids Rehabilitation Hospital

SAMPLE

Lokomat Session Log

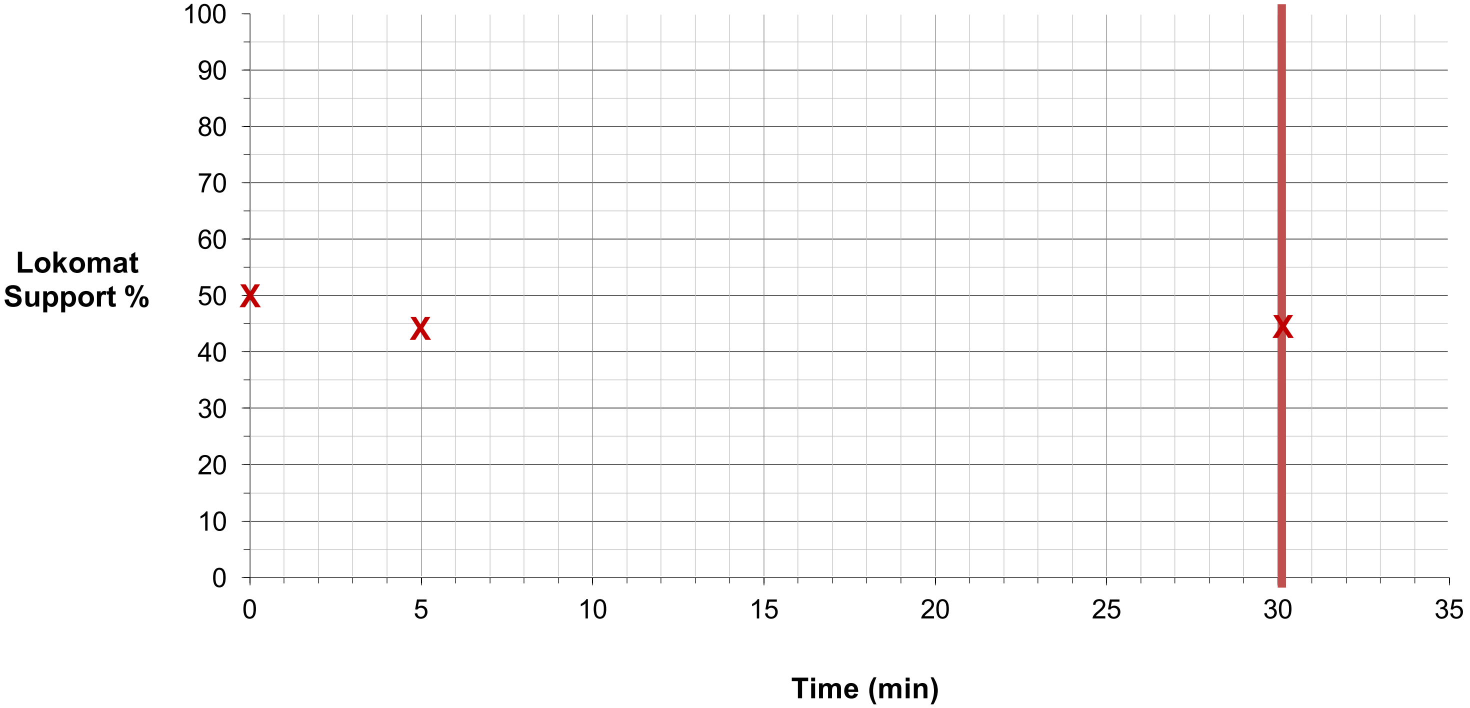

Remarks on Support:

Fill in details of why support was adjusted.
(i.e.) Support decreased from 50% to 45% once client warmed up. Support was maintained at 45% to support changes in speed and guidance force.

Total Distance (m): 761.1 Total Duration (min) including mid-session stops/free rides: 31:15

Number of stop/restarts: 2 Number of free rides: 0

Comments about stop/restarts or other Lokomat training issues:

$S_{1:}$ Stop at 15mins for heart rate and exertion scale.

$S_{2:}$ Stop at 28mins for heart rate and exertion scale.

Don't Forget to mark an X at child's start level (Y axis) for support, guidance and speed. When any of these are changed, mark the new level with an X. Mark a vertical line down all graphs to indicate end of session.

Hilderley, Fehlings, Lee, Wright. Holland Bloorview Kids Rehabilitation Hospital, Toronto, Canada, 2016
