# Supplementary material for: Comparison of a robotic-assisted gait training program with a program of functional gait training for children with cerebral palsy: design and methods of a two group randomized controlled cross-over trial
Source: Springerplus. 2016 Oct 28;5(1):1886. doi: 10.1186/s40064-016-3535-0 (PMC5084143; doi:10.1186/s40064-016-3535-0)
Supplement: Supplementary file 3 — Additional file 3. Physiotherapy Protocol. A detailed outline on content and reporting procedures for physiotherapy treatment arm. [file 40064_2016_3535_MOESM3_ESM.pdf]

Physiotherapy Protocol

FORMAT OF SESSION

Each PT session will be a **maximum of 35 minutes** in duration in addition to 10 introductory minutes of conversation/pain form completion/goal review/planning at the start of each session, and 5 minutes at the end of the session to wrap-up. (Thus, a total of about 50 minutes.)

UNDERLYING TRAINING PRINCIPLES

1. Treatment components should be selected to be **in line with the goals of the child and parent(s)** as articulated in the COPM/GAS plan. Use of individual goals may **increase the child's motivation**. Clearly outline how treatment components will help them achieve the outlined goals.
2. Continually think about how to **progressively increase the challenge**. Push the child to work to their energy and ability limit to enhance both the fitness training potential and motor learning. Build on accomplishments of previous sessions by increasing task difficulty.
3. **Structure practice to be random** rather than blocked, by interspersing different activities within the practice session. Introduce a **variety of movement** with activities and repetitions. The list on the next page provides a general menu of intervention areas that are permitted.
4. Practice continuous tasks, such as walking, in **whole practice** rather than part by breaking the skill down into components. Rather, practice the whole skill with a focus on developing specific or challenging elements. Remember to design skill practice in a manner that will **transfer to daily tasks**.
5. When beginning the treatment arm, give as much **verbal and visual feedback** as possible. Visual feedback can be provided using mirror and modelling, and physical guidance can be used when needed. Fade feedback as required to increase the challenge and to promote the child's reliance on **intrinsic feedback**. Draw the child's attention to intrinsic feedback by asking them to describe the feelings in his/her legs, or cueing the child to focus on specific sensations.
6. Provide opportunity for error and success. Encourage the child, and provide information about positive elements and areas to improve in both the performance of the task (i.e. movement quality) and outcome of the task (i.e. did the ball go in the hoop).

PT TRAINING

Any of the intervention areas outlined below can be chosen for a session. The same activities do not need to be done at each session. The length of time spent within each intervention area is at the discretion of the treating PT. The focus is likely a mix of several intervention areas.

Any basic work done on range of motion or stretching should not be part of the session as it is not part of an active motor learning-based program – if needed, take 10 to 15 minutes prior to the session to do this. Please record in the log if this time is added to any of the sessions.

When recording the focus of the intervention area on the PT treatment log sheet (see sample on last page), one or more categories can be used, e.g., exercises on the Bosu ball might be for strength, stability/balance and co-ordination. Each box on the first page of the log sheet should be marked to indicate this. The primary focus should be marked as '1' in the corresponding box, with all secondary categories marked as a '2'. You may change the primary focus of the same activity from session to session (e.g., if using the ball, the primary focus might be strength at one session and stability at the next). This is why it is important to complete the activity log with each session. You and your partner PT might choose to do different activities as well. This is fine as long as they line up with the child's goals, and have a logical link with the activities in the past sessions.

Don't forget: The time spent in each intervention and the specific details of the session (repetitions, weight, distance) must also be documented on the second page of the PT treatment log. This information is critical to guide you and your partner PT in progressions.

OVERGROUND WALKING PRACTICE
This is done within the context of the session as appropriate to the goal plan.

HOMEWORK
While we do not want you to give the children any additional physiotherapy exercises to work on during this trial, you can encourage them to practice the elements of gait they focused on during the physiotherapy session. Suggest they practice at home each day when they walk, and help them to select an environment in which to practice (i.e. hallway at home). Additionally, the children can be encouraged to do 'mind gym' work, when they spend time thinking about how to walk.

INTERVENTION AREAS

Intervention area	Components	GMFCS Level	
		II	III
**Functional strengthening**	Bosu ball, circuit training (strength stations)	X	X
**Isolated muscle strengthening**	Theraband work<br>Isometric positions<br>Core strength work	X	X
**Gait training (quality) ***	Barefoot work, focused gait pattern work, walking on different surfaces	X	X
**Endurance/Fitness Cardiovascular**	Laps on track (walk/run), walking high knees/big steps, circuit training, exercise bike, adapted trike, stair fitness, Wii – WiiSport/other VR, arm/leg ergometer	X	X
**Balance/stability work (higher level static)**	Bosu ball, balance beam, single leg balance, wobble board, Wii, dual tasks	X	X
**Balance/stability work (dynamic)**	Bosu ball, balance beam, stairs (hands/hands free), circuit training	X	X
**Treadmill training**	Maximum of 10 minutes – no extra support allowed	X	X
**Basic motor skill learning**	Transition/mobility skills on mat	X	X
**Basic motor skill learning**	Ambulation-based skills similar to the GMFM Stand and Walk skills		X
**Advanced motor skill practice**	Sports-based skill training, high level ball skills, circuit training	X	

Note –** ***no use **of Gait-Lite allowed**

Holland Bloorview
Kids Rehabilitation Hospital

SAMPLE

Child's Treatment Log for the PT Intervention:
PT Activity Tracking Table

Therapy intervention tracking table – Write each activity practiced during this session in the log. For each activity, place a '1' in box that indicates the focus of the activity. For all secondary areas of focus that the activity relates to in your session, place a '2' in the relevant boxes. The focus of an activity can change from session to session.

Activity	Duration (min)	Stability/ balance	Coordination	Strength	Gait (all aspects)	Fitness	Other (specify in the box)
BALANCING ON BOSU BALL	5	1		2			
STEP-UPS ON STAIRS	5		2	2	2	1	
BALANCE BEAM	5	1	2				
FITNESS CIRCUIT	10	2		2		1	Agility 2
EXERCISE BIKE	5			2		1	

Comments/clarification: *Add any notes* (i.e.) Note – this is a child who is working on balance, strength, agility and fitness rather than on specific gait quality improvements. As the child progresses, there might be a change in focus later in the intervention arm.

Holland Bloorview
Kids Rehabilitation Hospital

SAMPLE

Child's Treatment Log for the PT Intervention:
Activity Descriptions

Activity Descriptions – Provide details on activities listed above (repetitions, weight, distance)

Activity	Details
BALANCING ON BOSU BALL	*Describe the task, length of hold, number of repeats*
STEP-UPS ON STAIRS	*Indicate: # required, speed, any variations on the task*
BALANCE BEAM	*Describe what the task was and then any time or repetition or other parameters used*
FITNESS CIRCUIT	*Describe the circuit stations and any time or repetition or other parameters used at each one*
EXERCISE BIKE	*Indicate: time, resistance, distance covered, any other parameters*
