# Supplementary material for: Comparison of a robotic-assisted gait training program with a program of functional gait training for children with cerebral palsy: design and methods of a two group randomized controlled cross-over trial
Source: Springerplus. 2016 Oct 28;5(1):1886. doi: 10.1186/s40064-016-3535-0 (PMC5084143; doi:10.1186/s40064-016-3535-0)
Supplement: Supplementary file 4 — Additional file 4. Description of Measures used in the Trial. Details on outcome measures used in the trial including purpose and description of measure, rationale for use in trial, and summary of psychometric properties. [file 40064_2016_3535_MOESM4_ESM.pdf]

Holland Bloorview
Kids Rehabilitation Hospital

Additional file 4

Description of Measures used in the Trial

PRIMARY OUTCOME MEASURES	
**GMFM-66 (barefoot, no devices)** (Russell et al. 2013)	
Purpose	To evaluate gross motor capabilities in the areas of lying/rolling, sitting, crawling, standing and walking in children and youth with CP. Skills are foundational gross motor skills that typically-developing 5yr old child is capable of performing.
Description	An internationally used standard of assessment for gross motor skills of children with CP. The child performs a series of up to 37 Standing and Walking skills. This test is done barefoot.
Rationale for use in trial	Expected direct effect of Lokomat (LOK) training on gait-based gross motor skills. Gains of 3+ points consistently detected in single group LOK studies. Use as the co-primary measure allows direct comparison of our results with almost all other paediatric LOK studies.
Psychometric properties	**Reliability:** Good to excellent inter-rater reliability in CP and similar levels for test-retest reliability in CP (Russell et al. 2000). **Validity:** Strongly established for construct and discriminant validity in CP (Lundkvist Josenby et al. 2009). **Responsiveness:** Demonstrated by several international research groups as 4 points for the GMFM and 3 points the GMFM-66. Used extensively in clinical trials with children in GMFCS and appears to be a very effective measure of underlying change (Wang and Yang 2006).
**6 minute walk test (6MWT)** (Thompson et al. 2008)	
Purpose	To evaluate functional walking capacity in a clinical setting without the need for specialized equipment. Recommended as a submaximal exercise test for children in Gross Motor GMFCS I, II, or III that should be included in a ‘core set‘ of exercise tests for children with CP (Verschuren et al. 2011).
Description	Single scale; the total distance walked in 6 minutes with devices as needed. Can be used with children (≥ 4y) with wide range of impairments that could theoretically affect walking capacity. Have to be able to follow test directions and manage > 6 minute distance without person’s physical assistance.
Rationale for use in trial	Expected direct effect of LOK training on walking endurance due to treadmill component of LOK. Link between 6 minute walk times and endurance/ fitness. Use as the co-primary measure allows direct comparison of our results with almost all other pediatric LOK studies.
Psychometric properties	**Reliability:** Test-retest reliability with cerebral palsy in GMFCS III is excellent (Thompson et al. 2008; Maher et al. 2008). **Validity:** Convergent validity ($0.44 \leq r \leq 0.62$) with maximum oxygen uptake has been reported for typically developing children and for those with spina bifida. **Responsiveness:** In children with CP, the minimal detectable change is 16% (54.9 m) (Thompson et al. 2008).
**GMFM Stand & Walk/Run/Jump Dimensions (for aids/orthoses)** (Russell et al. 2000; Russell and Gorter 2005)	
Purpose	Children in GMFCS Level III will also do the Stand and Walk dimension items (up to15 items in total are likely possible for these children) with shoes/orthoses/aides since this better reflects functional performance than barefoot testing.
Description	Not Rasch scaled, so have to use Stand and Walk raw scores on their own to arrive at % score. Standard guidelines in the manual to administer the Stand and Walk items in this way.
Rationale for use in trial	Permits a look at gross motor changes that pertain more closely to usual means of mobility (devices and orthoses). Functional relevance and also allows child to do some of the items that could not do barefoot due to stability issues. Particularly useful with kids in GMFCS III who rely on devices.
Psychometric properties	**Reliability:** Less known about reliability. Assumed to be as for barefoot test. **Validity:** Strong correlation with timed walk test. **Responsiveness:** Not studied specifically but has been able to detect change in children who have received a walking based intervention (Russell and Gorter 2005).

SECONDARY OUTCOME MEASURES	
**Timed Up and Go (TUG)** (Williams et al. 2005)	
Purpose	Assesses functional mobility, balance and postural control through execution of an integrated movement sequence.
Description	Items consist of standing from a chair, walking three meters and returning to the chair. Shorter times indicate higher functional ability.
Rationale for use in trial	Detection of improvements in balance and functional movement that is appropriate for measurement in GMFCS Levels II-III. Will permit comparison to other LOK trials that use this measure. A walk distance of 5-meters will be used in this trial to increase sensitivity to change.
Psychometric properties	**Reliability:** High within-session reliability and same-day retest in children with CP in GMFCS Levels I-III. **Validity:** moderate negative correlations with GMFM Stand and Walk dimension items. **Responsiveness:** Established in typically developing children (Williams et al. 2005); sensitivity to change in children with CP comparable to GMFM (de Campos et al. 2011).
***Challenge* Assessment** (Wilson et al. 2011)	
Purpose	This test was developed for high functioning children to capture changes in advanced motor skills that the GMFM is not sensitive to.
Description	A 25-item tool with items to evaluate performance in more advanced motor skills requiring balance, speed, and coordination. Items are scored by a PT on a scale of 0-4. Children have three chances to perform the skill (Wilson et al. 2011; Glazebrook and Wright 2014).
Rationale for use in trial	Children in GMFCS Level II may show improvement in advanced skills that are not captured by the GMFM.
Psychometric properties	**Reliability:** Evidence of strong inter-rater and test-retest reliability in a pilot study with 16 children in GMFCS I (Wright et al. 2012). **Validity:** Not reported. **Responsiveness:** Not reported.
**Quality Function Measure (QFM)** (Wright et al. 2014)	
Purpose:	To evaluate key aspects of quality related to standing walking and running skills.
Description	This measure is rated from the child's GMFM-66 video. The child does not have to do any extra testing. Scoring is by a trained PT from the video looking at the 5 attributes of alignment, coordination, dissociated movement, stability and weight shift.
Rationale for use in trial	This will provide insight into important changes in quality of movement, which may not be reflected in quantitative testing.
Psychometric properties	**Reliability:** Demonstrated to be excellent (inter-rater and test-retest) in a sample of children with CP in GMFCS I, II and III (Wright et al. 2014). **Validity:** Good discriminant validity among GMFCS Levels (Wright et al. 2014). **Responsiveness:** Evaluation underway by senior author (V Wright). Results are promising as far as detection of change post-Botox or orthopedic surgery.
**Gait Kinematics using GAITRite® walkway system** (Sorsdahl et al. 2008)	
Purpose	To evaluate time-distance parameters of gait (velocity, cadence, step/stride length, width).
Description	A roll-out 10 m long mat captures footfall, gait cycle and allows PC software analysis. Children will conduct a repeat of 6 walks along the mat, taking average of middle 4 trials. A 3m lead/follow-up distance will control for acceleration/deceleration (Klejman et al. 2010). Trials will be performed with shoes and AFOs to replicate functional gait.
Rationale for use in trial	Expected direct effect of LOK training given focus on quality of gait and treadmill training component. This is a common measure in other LOK trials so allows comparison of our results with others.
Psychometric properties	**Reliability and Validity:** Strong in adults (Webster et al. 2005). Expect as per VICON assessment given same walk procedure used (Klejman et al. 2010). Reliability of cadence, step length, stride length and single stance time was high to excellent in children with CP (Sorsdahl et al. 2008). **Responsiveness:** 95% MDC values for GAITRite more responsive to change than observation measures in older adults (Youdas et al. 2010).

**Observational Gait Assessment using the Holland Bloorview Observational Gait Scale** (Wright et al. 2008)	
Purpose	To evaluate quality of gait in terms of components of the gait cycle.
Description	This is a 30-item measure developed by members of our investigative team specifically for use with children with CP who are undergoing an intervention that is hoped to have an impact of gait quality (e.g., botulinum toxin, gait-based PT, orthopaedic surgery). Scored from a lateral/anterior posterior video view video of the child's walking along a 15 m pathway.
Rationale for use in trial	Potential direct effect of LOK training on quality components of gait, e.g., better knee extension in stance, greater hip extension through push-off, better heel contact at heel strike. Readily scored (5-minutes viewing per video) and gives more of an dynamic quality of movement focus than other observational measures, so hopefully more able to pick up functionally relevant gait changes.
Psychometric properties	**Reliability**: Demonstrated to be excellent (inter-rater and test-retest) in a sample of children with CP in GMFCS I, II and III (Wright et al. 2008). **Validity:** Good discriminant validity among GMFCS Levels (Wright et al. 2008). **Responsiveness:** Not tested.
**Pediatric Evaluation of Disability (PEDI) Caregiver Assistance** (Haley et al. 1992)	
Purpose	For more involved children, PEDI assesses the ability of the child to perform Functional Skills in their own environment (Part 1), and establishes the level of Caregiver Assistance required (Part 2).
Description	A parent-reported measure. Functional skills scale assesses 97 skills on a dichotomous scale of 'can'/'cannot' perform. The Caregiver Assistance scale ranks independence of the child in performing skills from 0 (total assistance) to 5 (independent).
Rationale for use in trial	PEDI Part II provides a means to measure the impact of LOK and PT on a child's independence in daily tasks. Gross motor based skills covered instead via the ASK-30.
Psychometric properties	**Reliability**: High internal consistency and good to excellent inter-rater and test-retest reliability (Haley et al. 1992; Berg et al. 2004). **Validity**: Excellent construct validity. **Responsiveness:** No ceiling or floor effects detected (Vos-Vromans et al. 2005).
**Activity Scale for Kids (ASK)-30** (performance version) (Young et al. 2000)	
Purpose	This questionnaire gives us a picture of how a child moves at home and school by evaluating the ability of the child to perform (a reflection of degree of difficulty) a set of 30 daily activities.
Description	Client-report (child 8y+/parent of younger child) questionnaire. Thirty questions with 5-point response scale (able to do all of the time to none of the time). Can be supplemented to cover more sports-based motor skills with eight additional questions by Bagley et al. (2011).
Rationale for use in trial	Provides an opportunity to look at any impact of LOK or PT on day-to-day mobility- and self-care based skills at home and school. The use of standard items will allow comparison across children and is then available to consider in the light of individualized goal accomplishment. ASK results also allow comparison to numerous other PT studies that have used the ASK as an outcome measure. Will report both the ASK-30 and additional 8-item scores. In this trial if child is 8y+, both child and parent will complete the questionnaire.
Psychometric properties	**Reliability:** ASK-30 well established by the developers for children with neuromuscular conditions (Young et al. 2000). **Validity:** Well established by the developers for children with neuromuscular conditions (Young et al. 2000). **Responsiveness:** Not directly known; thought to be about 3 points. Used extensively in clinical research in CP and other NM conditions.
**Children's Assessment of Participation and Enjoyment** (**CAPE**) (Physical Activity sections items 16 –21 and 31-41) (King et al. 2007)	
Purpose	To evaluate the types of activities a child participates, the extent to which they participate and how much they enjoy the activity.
Description	Client-report (child 8y+/parent of younger child) questionnaire. Child responds to whether they have performed the activity in the last few weeks, who they did it with, where they did it and how much they enjoyed it in a multiple choice format. Can use sections or the entire form.
Rationale for use in trial	Able to use subcategories organized sports (items 16-21), and active physical recreation (items 31-41) to keep the focus on physical activity(Imms 2008; Scholtes et al. 2008). In this trial if child is 8y+, both child and parent will complete the questionnaire.
Psychometric properties	**Reliability:** Strong internal consistency and test-retest (Imms 2008; Capio et al. 2010). **Validity:** Construct validity established (King et al. 2007; Imms 2008). **Responsiveness:** Some evidence now in use within clinical trials that able to detect change. No specific responsiveness studies done though and no estimates of important change yet.

**KIDSCREEN-27 (QOL)** (Ravens-Sieberer et al. 2007)	
Purpose	This short form questionnaire assesses a child's health and well-being and satisfaction with life, as reported from the child's perspective.
Description	27 items scale evaluating five Rasch-scaled dimensions of Physical Well-Being, Psychological Well-Being, Parents & Autonomy, Social Support & Peers, School Environment.
Rationale for use in trial	KIDSCREEN-27 permits investigation into the possible relationship LOK and PT may have on improving QOL. In this trial if child is 8y+, both child and parent will complete the questionnaire.
Psychometric properties	**Reliability**: Acceptable internal consistency and test-rest reliability (Ravens-Sieberer et al. 2007). **Validity**: Construct and criterion validity established in TD children (Ravens-Sieberer et al. 2007). Items in KIDSCREEN-52 interpreted the same by children with CP and TD (Erhart et al. 2009). **Responsiveness:** Not investigated.
**Canadian Occupational Performance Measure (COPM)** (Law et al. 1990)	
Purpose	To identify and subsequently evaluate outcomes related to a child's individualized goals in the areas of self-care, productivity and leisure. The COPM is used extensively in pediatric practice and research.
Description	An interview style measure in which child is asked by assessor to identify goal areas and then rate their current level on a 1 to 10 scale as far as importance, satisfaction and performance. Typically elicit three to five goals areas for a block intervention. Standard COPM interview approach and form used.
Rationale for use in trial	Functional therapy programs need to be directed toward the child's and parent's goals to maximize value of outcomes and engagement. Use of individualized goals has been missing from all previous LOK studies. Information gleaned will help us to understand the potential of the LOK and PT for achieving meaningful outcomes and will also give us guidelines for future practice as far as types of goals that tend to be associated with these therapies.
Psychometric properties	**Reliability:** Test-retest reliability is strong. Inter-rater reliability N/A given style of test (Law et al. 1990). **Validity**: Multiple studies showing strong construct validity (Law et al. 1990). **Responsiveness**: Changes of 2 points on the COPM are considered to be clinically important and are very realistic as far as possibilities with effective interventions (Law et al. 1990; Tam C et al. 2008).
**Goal Attainment Scaling (GAS)** (King et al. 2000; McDougall and Wright 2009)	
Purpose	To identify and subsequently evaluate a child's individualized goals in any area of impairment, activity, participation or QOL. Complements the COPM as it requires the rating of observable behaviours.
Description	This is a 5-point rating system (-2 to +2) in which the child's current abilities are described and recorded as the -2 (starting point). The aim is to improve the abilities that are targeted to achieve a level of 0 on the GAS form. -1, +1 and +2 represent differing extents of achievement and allow a clear operationalization of the abilities acquired.
Rationale for use in trial	A practical approach to goal setting in PT and OT in which goals tend to be quite easily scaled according to observable behaviours. When used with the COPM for a similar set of goals, give context as to what the exact abilities are that tie in with the ratings of satisfaction and performance.
Psychometric properties	**Reliability:** Test-retest reliability is strong. Inter-rater reliability N/A given style of test (McDougall and Wright 2009). **Validity:** Multiple studies showing strong construct validity (McDougall and Wright 2009). **Responsiveness:** Numerous trials in pediatric rehabilitation have shown that children accomplish the level 0 goals (indication of change) (McDougall and Wright 2009).

OTHER MEASURES	
**Dimension of Mastery Questionnaire** (Igoe et al. 2011; Miller et al. 2014)	
Purpose	To measure a child's motivation mastery (ability to persevere, stick with difficult tasks, feel successful, etc.)
Description	Client-report (child 8y+/parent of younger child) questionnaire. A series of 45 questions and a 5-point "not at all like you" to "very much like you" scale.
Rationale for use in trial	While this has not been studied before with LOK or PT, clinicians tell us that kids who are more prepared to keep trying and respond to/recognize small gains may have better outcomes. This could be especially the case with motor learning based interventions in which engagement is critical. In this trial if child is 8y+, both child and parent will complete the questionnaire.
Psychometric properties	**Reliability:** Evaluated with young children with CP and those of school age with evidence of good to strong reliability (Igoe et al. 2011; Miller et al. 2014). **Validity:** Not tested. **Responsiveness:** N/A. Likely a trait, not something that changes.
**Pain Combined use of FACES Scale** (Wong and Baker 1988; Garra et al. 2010; Tomlinson et al. 2010) **for specific pain areas and a generic body diagram to show areas of pain**	
Purpose	To measure a child's current pain specific to body areas.
Description	Client-report questionnaire (all ages). Uses child-based body diagrams and faces scales of pain to allow easy reporting.
Rationale for use in trial	Essential from the perspective of the LOK therapy to be aware at all times of the presence and degree of any related pain.
Psychometric properties	**Reliability:** Well-established scales for children and youth. **Validity:** Established. **Responsiveness:** Not tested but demonstrated in practice (Wong and Baker 1988; Garra et al. 2010; Tomlinson et al. 2010).
**ROM/spasticity (Tardieu)** (Boyd and Graham 1999; Scholtes et al. 2006)	
Purpose	To measure active and passive joint range (ROM) and to evaluate passive and dynamic spasticity.
Description	ROM – goniometric measure according to standardised positions for children with CP.<br>Tardieu – slow and fast stretch of dorsiflexors and hamstrings to elicit initial resistance to dynamic stretch (R1) and end range resistance (R2).
Rationale for use in trial	Part of the monitoring of range and spasticity to ensure safety of the treatments and detection of any deterioration.<br>Will help to answer question about impact of LOK and PT on ROM.
Psychometric properties	**Reliability:** Goniometry well-established in children with CP as long as standard position and same tester used Tardieu inter-rater and test-retest reliability fair to excellent in CP (Boyd and Graham 1999; Scholtes et al. 2006).**Validity:** Strong for ROM. Assumed, not tested for Tardieu. **Responsiveness:** Changes of 15 + degrees required to show true change.
**Pictorial Children's Effort Rating Scale (PCERT)** (Yelling et al. 2002) **and Heart Rate**	
Purpose	RPE: To measure how hard the child feels that he/she is working while doing an activity.<br>Heart rate: To obtain an objective, easy to measure value reflecting physiological effort.
Description	PCERT: Self-report assessment using a 1-10 point ladder scale rating from "very, very easy" to "so hard I'm going to stop (Yelling et al. 2002); Heart rate will be measured in beats per minute via radial pulse during the LOK and PT sessions (#/15sec x 4) as too difficult to apply HR tracking instrumentation when child is in the LOK.
Rationale for use in trial	Measures reflecting physiological effort will ensure that the children are actively involved in the session (LOK and PT) and not in the free ride mode on the LOK. Use at the mid and end points of the session allow the PT to get a sense of the child's effort and coach accordingly
Psychometric properties	**Reliability and validity:** PCERT highly correlated with quantitative measures of exercise intensity in children with strong test-retest (Marinov and Mandadjieva 2008). **Responsiveness:** Not reported.

**Physical Activity Enjoyment Scale (PACES)** (Moore et al. 2009)	
Purpose	To evaluate what children think about the treatment and how it makes them feel.
Description	A self-report 16-item measure ranking feelings when being physically active on a 5-point Likert scale.
Rationale for use in trial	In addition to giving us feedback on the acceptability of each intervention from the child's viewpoint, it will also be possible to evaluate changes in the child's response to being active. There is evidence that activity can provide a sense of well-being (Hardy and Rejeski 1989), a potentially important outcome in itself as it may contribute to an individual's desire to become more physically active and reduce potentially harmful sedentary behaviours (Owen et al. 2010). A 10-item modified version of the PACES is used in this trial so that items evaluated make sense in the context of the Lokomat/PT sessions.
Psychometric properties	**Reliability:** High internal consistency in TD children and good item-total correlations. **Validity:** Convergent validity established (Moore et al. 2009). **Responsiveness:** Not tested.
**Activity Monitor** (Song et al. 2006)	
Purpose	The StepWatch™ is a commercially available accelerometer designed for patients with mobility issues, sensitive to abnormal step patterns and able to record steps over an extended period of time (typically 1-2weeks) and provide information on step frequency (Song et al. 2006).
Description	A StepWatch will be worn at home over a 5 day period including over a weekend to the child's determine usual walking activity. The total step count per day and the mean of the 5 days will be used in the data analysis. This will be done in the pre-intervention period (baseline), and at the 8-week point of each intervention, and in the 4th week of the 6-week wash-out phase.
Rationale for use in trial	The StepWatch will provide data on the habitual activity frequency and level of the child in his/her natural environment (Bjornson et al. 2014b), and permit analysis of change following treatment.
Psychometric properties	**Reliability:** High reliability in children with CP (Song et al. 2006). **Validity:** High criterion validity against manual step count in typically developing children (TDC) (Bjornson et al. 2012) and strong discriminatory ability between TDC and children with CP, as well as GMFCS level (Stevens et al. 2010; Bjornson et al. 2014a; Bjornson et al. 2014b). **Responsiveness:** Not tested.

*Details for several measures excerpted from Wright et al. (2013) and Sanders et al. (2013)

References

Bagley AM, Gorton G, Bjornson K, et al (2011) Factor- and item-level analyses of the 38-item activities scale for kids-performance. Dev Med Child Neurol 53:161–166.
Berg M, Jahnsen R, Frøslie KF, Hussain A (2004) Reliability of the Pediatric Evaluation of Disability Inventory (PEDI). Phys Occup Ther Pediatr 24:61–77.
Bjornson KF, Yung D, Jacques K, et al (2012) StepWatch stride counting: accuracy, precision, and prediction of energy expenditure in children. J Pediatr Rehabil Med 5:7–14. doi: 10.3233/PRM-2011-0186
Bjornson KF, Zhou C, Stevenson R, et al (2014a) Walking activity patterns in youth with cerebral palsy and youth developing typically. Disabil Rehabil 36:1279–1284. doi: 10.3109/09638288.2013.845254
Bjornson KF, Zhou C, Stevenson RD, Christakis D (2014b) Relation of Stride Activity and Participation in Mobility-Based Life Habits Among Children With Cerebral Palsy. Arch Phys Med Rehab 95:360–368. doi: 10.1016/j.apmr.2013.10.022
Boyd RN, Graham HK (1999) Objective measurement of clinical findings in the use of botulinum toxin type A for the management of children with cerebral palsy. Eur J Neurol 6:s23–s35. doi: 10.1111/j.1468-1331.1999.tb00031.x
Capio CM, Sit CH, Abernethy B, Rotor ER (2010) Physical activity measurement instruments for children with cerebral palsy: a systematic review. Dev Med Child Neurol 52:908–916.
de Campos AC, Costa CSND, Rocha NACF (2011) Measuring changes in functional mobility in children with mild cerebral palsy. Dev Neurorehabil 14:140–144. doi: 10.3109/17518423.2011.557611
Erhart M, Ravens-Sieberer U, Dickinson HO, Colver A (2009) Rasch measurement properties of the KIDSCREEN quality of life instrument in children with cerebral palsy and differential item functioning between children with and without cerebral palsy. Value Health12:782–792. doi: 10.1111/j.1524-4733.2009.00508.x

Garra G, Singer AJ, Taira BR, et al (2010) Validation of the Wong-Baker Faces Pain Rating Scale in pediatric emergency department patients. Acad Emerg Med 17:50–54.

Glazebrook CM, Wright FV (2014) Measuring advanced motor skills in children with cerebral palsy: further development of the Challenge module. Pediatr Phys Ther 26:201–213. doi: 10.1097/PEP.0000000000000035

Haley SM, Coster WJ, Ludlow LH, et al (1992) Pediatric Evaluation of Disability Inventory (PEDI). Pearson Clinical, Texas

Hardy CJ, Rejeski WJ (1989) Not What, But How One Feels: The Measurement of Affect During Exercise. J Sport Exerc Psychol 11:304–317.

Igoe D, Peralta C, Jean L, et al (2011) Evaluation of the test-retest reliability of the Dimensions of Mastery Questionnaire (DMQ) in preschool-aged children. Infants Young Child 24:280–291.

Imms C (2008) Review of the children's assessment of participation and enjoyment and the preferences for activity of children. Phys Occup Ther Pediatr 28:389–404.

King GA, Law M, King S, et al (2007) Measuring children's participation in recreation and leisure activities: construct validation of the CAPE and PAC. Child Care Health Dev 33:28–39. doi: 10.1111/j.1365-2214.2006.00613.x

King GA, McDougall J, Palisano RJ (2000) Goal attainment scaling: its use in evaluating pediatric therapy programs. Phys Occup Ther Pediatr 19:31–52. doi: 10.1080/J006v19n02_03

Klejman S, Andrysek J, Dupuis A, Wright V (2010) Test-retest reliability of discrete gait parameters in children with cerebral palsy. Arch Phys Med Rehab 91:781–787. doi: 10.1016/j.apmr.2010.01.016

Law M, Baptiste S, McColl M, et al (1990) The Canadian occupational performance measure: an outcome measure for occupational therapy. Can J Occup Ther 57:82–87.

Lundkvist Josenby A, Jarnlo G-B, Gummesson C, Nordmark E (2009) Longitudinal construct validity of the GMFM-88 total score and goal total score and the GMFM-66 score in a 5-year follow-up study. Phys Ther 89:342–350. doi: 10.2522/ptj.20080037

Maher CA, Williams MT, Olds TS (2008) The six-minute walk test for children with cerebral palsy. Int J Rehabil Res 31:185–188. doi: 10.1097/MRR.0b013e32830150f9

Marinov B, Mandadjieva S (2008) Pictorial and verbal category-ratio scales for effort estimation in children. Child: care. doi: 10.1111/j.1365-2214.2007.00767.x/full

McDougall J, Wright V (2009) The ICF-CY and Goal Attainment Scaling: Benefits of their combined use for pediatric practice. Disabil Rehabil 31:1362–1372. doi: 10.1080/09638280802572973

Miller L, Marnane K, Ziviani J, Boyd RN (2014) The Dimensions of Mastery Questionnaire in school-aged children with congenital hemiplegia: test-retest reproducibility and parent-child concordance. Phys Occup Ther Pediatr 34:168–184. doi: 10.3109/01942638.2013.806978

Moore JB, Yin Z, Hanes J, et al (2009) Measuring Enjoyment of Physical Activity in Children: Validation of the Physical Activity Enjoyment Scale. J Appl Sport Psychol 21:S116–S129. doi: 10.1080/10413200802593612

Owen N, Healy GN, Matthews CE, Dunstan DW (2010) Too much sitting: the population health science of sedentary behavior. Exerc Sport Sci Rev 38:105–113. doi: 10.1097/JES.0b013e3181e373a2

Ravens-Sieberer U, Auquier P, Erhart M, et al (2007) The KIDSCREEN-27 quality of life measure for children and adolescents: psychometric results from a cross-cultural survey in 13 European countries. Qual Life Res 16:1347–1356. doi: 10.1007/s11136-007-9240-2

Russell DJ, Rosenbaum PL, Wright M, Avery LM (2013) Gross Motor Function Measure (GMFM-66 and GMFM-88) user’s manual, 2nd edn. Wiley, Hoboken

Russell DJ, Gorter JW (2005) Assessing functional differences in gross motor skills in children with cerebral palsy who use an ambulatory aid or orthoses: can the GMFM-88 help? Dev Med Child Neurol 47:462–467.

Russell DJ, Wright M, Rosenbaum PL, et al (2000) Improved scaling of the Gross Motor Function Measure for children with cerebral palsy: evidence of reliability and validity. Phys Ther 80:873–885.

Sanders H, Wright V, Burtner P (2013) Mobility. In: Majnemer A (ed) Measures for Children with Developmental Disability framed by the ICF-CY. London, pp D410–D489

Scholtes VA, Becher JG, Beelen A, Lankhorst GJ (2006) Clinical assessment of spasticity in children with cerebral palsy: a critical review of available

instruments. Dev Med Child Neurol 48:64–73. doi: 10.1017/S0012162206000132
Scholtes VA, Dallmeijer AJ, Rameckers EA, et al (2008) Lower limb strength training in children with cerebral palsy--a randomized controlled trial protocol for functional strength training based on progressive resistance exercise principles. BMC Pediatr 8:41. doi: 10.1186/1471-2431-8-41
Song KM, Bjornson KF, Cappello T, Coleman K (2006) Use of the StepWatch Activity Monitor for Characterization of Normal Activity Levels of Children. J Pediatr Orthop 26:245–249.
Sorsdahl AB, Moe-Nilssen R, Strand LI (2008) Test-retest reliability of spatial and temporal gait parameters in children with cerebral palsy as measured by an electronic walkway. Gait Posture 27:43–50. doi: 10.1016/j.gaitpost.2007.01.001
Stevens SL, Holbrook EA, Fuller DK, Morgan DW (2010) Influence of age on step activity patterns in children with cerebral palsy and typically developing children. Arch Phys Med Rehab 91:1891–1896. doi: 10.1016/j.apmr.2010.08.015
Tam C, Teachman G, Wright V (2008) Paediatric Application of Individualised Client-Centred Outcome Measures: a Literature Review. Br J Occup Ther 71:286–296.
Thompson P, Beath T, Bell J, et al (2008) Test-retest reliability of the 10-metre fast walk test and 6-minute walk test in ambulatory school-aged children with cerebral palsy. Dev Med Child Neurol 50:370–376. doi: 10.1111/j.1469-8749.2008.02048.x
Tomlinson D, Baeyer Von CL, Stinson JN, Sung L (2010) A systematic review of faces scales for the self-report of pain intensity in children. J Pediatr 126:e1168–1198.
Verschuren O, Ketelaar M, Keefer D, et al (2011) Identification of a core set of exercise tests for children and adolescents with cerebral palsy: a Delphi survey of researchers and clinicians. Dev Med Child Neurol 53:449–456. doi: 10.1111/j.1469-8749.2010.03899.x
Vos-Vromans DCWM, Ketelaar M, Gorter JW (2005) Responsiveness of evaluative measures for children with cerebral palsy: the Gross Motor Function Measure and the Pediatric Evaluation of Disability Inventory. Disabil Rehabil 27:1245–1252. doi: 10.1080/09638280500076178
Wang H-Y, Yang YH (2006) Evaluating the responsiveness of 2 versions of the gross motor function measure for children with cerebral palsy. Arch Phys Med Rehabil 87:51–56. doi: 10.1016/j.apmr.2005.08.117
Webster KE, Wittwer JE, Feller JA (2005) Validity of the GAITRite® walkway system for the measurement of averaged and individual step parameters of gait. Gait Posture 22:317–321.
Williams EN, Carroll SG, Reddihough DS, et al (2005) Investigation of the timed “Up & Go” test in children. Dev Med Child Neurol 47:518–524. doi: 10.1017/S0012162205001027
Wilson A, Kavanaugh A, Moher R, et al (2011) Development and pilot testing of the Challenge Module: a proposed adjunct to the Gross Motor Function Measure for high functioning children with cerebral palsy. Phys Occup Ther Pediatr 31:135–149. doi: 10.3109/01942638.2010.489543
Wong DL, Baker CM (1988) Pain in children: comparison of assessment scales. Pediatr Nurs 14:9–17.
Wright FV, Rosenbaum P, Fehlings D, et al (2014) The Quality Function Measure: reliability and discriminant validity of a new measure of quality of gross motor movement in ambulatory children with cerebral palsy. Dev Med Child Neurol 56:770–778. doi: 10.1111/dmcn.12453
Wright V, Maltais DB, Sanders H, Burtner P (2013) Measures of outcomes and their determinants for children and youth with developmental disabilities. In: Majnemer A (ed) Measures of outcomes and their determinants for children and youth with developmental disabilities. Mac Keith Press, London UK, pp B710–780
Wright V, Redekop S, Koo I (2008) Reliability of a new observational gait scale for evaluation of outcomes related to botulinum toxin type-A injections in children with cerebral palsy. Dev Med Child Neurol 50:S32.
Wright V, Shircore LE, Fehlings D, Lee G (2012) Reliability of the Challenge Module for children with cerebral palsy in GMFCS Level I. Dev Med Child Neurol 54:30–79. doi: 10.1111/j.1469-8749.2012.04388.x
Yelling M, Lamb KL, Swaine I (2002) Validity of a pictorial perceived exertion scale for effort estimation and effort production during stepping exercise in adolescent children. Eur Phys Educ Rev 8:157–175. doi: 10.1177/1356336X020082007
Youdas JW, Childs KB, McNeil ML, et al (2010) Responsiveness of 2 Procedures for Measurement of Temporal and Spatial Gait Parameters in Older Adults. PM&R 2:537–543. doi: 10.1016/j.pmrj.2010.02.008
Young NL, Williams JI, Yoshida KK, Wright JG (2000) Measurement properties of the activities scale for kids. J Clin Epidemiol 53:125–137. doi: 10.1016/S0895-4356(99)00113-4
